# Comparison of the specific energies of sinusoidal VCS cutter rings and CCS cutter rings in breaking rock-like materials based on the FEM

**DOI:** 10.1038/s41598-024-58466-0

**Published:** 2024-04-07

**Authors:** Jia Li Zhao, Xian Yong Zhu, Hui Zhang, Hua Liang Xu, Song Yang, Peng Wu, Xiang Mi You

**Affiliations:** 1 School of Mechanical and Aerospace Engineering, Jilin University, 5988 Renmin StreetNanguan District, Changchun, 130000 Jilin Province China; 2 School of Aeronautical Engineering, Jilin Institute of Chemical Technology, Jilin, 132012 China; 3 Northeast Electric Power University, Jilin, 132012 China; 4 Changchun Baoze Technology Co., Ltd., Changchun, 130051 China; 5 CISDI GROUP Co., Ltd., Chongqing, 401122 China

**Keywords:** Disc cutter, Rock breaking, Finite element method, VCS, CCS, Mechanical engineering, Petrology

## Abstract

Disc cutters are essential for full-section hard-rock tunnel boring machines. The performance of these devices directly affects tunnel engineering costs and duration. This paper proposes a sinusoidal variable cross-section (VCS) cutter ring and design method and establishes a digital model. Rock-like materials are simulated with a finite element model, and the model validity is verified via rock simulation mechanics tests. A disc cutter rolling rock simulation model for a linear cutting machine is also established, and simulation tests are performed for single- and three-cutter rolling using sinusoidal VCSs and constant cross-section (CCS) cutter models, respectively. The stress and energy changes for the cutters and rock-like material damage area were compared via simulation, confirming that some sinusoidal VCS cutter rings do less work on rock-like materials and cause larger crushing areas under the same engineering parameters; therefore, these cutter rings have smaller specific energies. The sinusoidal VCS cutter ring performance is 7% greater than that of CCS on average under single-cutter simulation, and the intermediate cutter performance of the intermediate cutter is 9% greater than that of CCS on average under three-cutter simulation. Thus, sinusoidal VCS cutter rings offer improved rock damage performance, and further research and application of this technology will improve the working efficiency of tunnel boring machines.

## Introduction

A tool system is highly important for hard rock tunnel boring machines (TBMs), and disc cutter performance is directly related to the engineering efficiency and reliability of TBMs^1^. During the boring process, the cutter head rotates and drives the disc cutter to roll into the rock and peel chips from the rock body.

Therefore, the disc cutter is in direct contact with the rock and is the core component for breaking the rock^2^. Improving the disc cutter rock breaking capability is crucial for increasing the tunneling speed and reducing the construction cost^3^. Many previous studies have investigated improving disc cutter performance from many aspects.

In the theoretical research on rock breaking using disc cutters, Rostami^4^ proposed a theory for the interaction between disc cutters and rock, providing a mature rock breaking theory. Bruland^5^ subsequently analyzed the rock breaking process using plastic and elastic‒plastic mechanics and other methods, providing an important framework for optimizing cutter design and improving rock breaking efficiency. Maji and Theja^6^ proposed a theoretical model for rock failure based on rock uniaxial compressive strength and rock failure surface shape.

In the past decade, research on the performance of disc cutters has adopted mainly the method of mechanical testing by testing machines; however, the experimental equipment is expensive, the experimental process is complex, and only a few countries in the world have full-size disc cutter rolling rock testing machines, including the United States, China, Korea and other countries. Most experimental studies use linear cutting machines (LCMs) because they can strictly control engineering parameters, including tool spacing, penetration, thrust and speed, to minimize uncertainties due to size effects. Full-scale LCM tests have been widely used to predict TBM cutter performance^7–13^. Several LCM rock breaking experiments have also been conducted at Central South University^14–16^ to help describe load variations as a CCS disc cutter breaks the rock mass and establish functional relationships between cutter geometric parameters and force.

With the development of computer technology, an increasing number of scholars have used computer simulation technology to study the performance of disc cutters. The finite element method (FEM) and discrete element method (DEM) have been widely used in many fields related to disc cutters. Xia et al.^17^ used AUTODYN to simulate disc cutter rock breaking under free surface conditions and derived relationships between cutter machine load responses. Xiao et al.^18^ proposed a rock material constitutive model coupled with the FEM using Abaqus, considering dynamic rock breaking load changes. Moon et al.^19^ and Choi et al.^20^ established rock breaking simulation models using DEMs for different tool structures and geological parameters and subsequently investigated load and energy consumption changes under different working conditions. In recent years, an increasing number of simulation studies have been performed ^21–23^, which more intuitively reveal some phenomena in the rock breaking process of disc cutters and contribute to the progress of TBM technology.

This paper proposes a design method for sinusoidal cutter rings with variable cross sections (VCSs) considering rock mechanical properties and subsequently predicts the cutter ring performance. An FEM for rock-like materials is established, and rock mechanics experiments are performed to verify the validity of the model. Rock breaking processes for sinusoidal VCSs and CCS cutter rings are also simulated and compared for LCM test principles, particularly considering the cutter ring force and energy and the rock crushing volume. Several sinusoidal VCS cutter rings were identified that consumed less energy during the rock breaking process, with a larger broken rock volume, i.e., less specific energy required and hence higher rock breaking efficiency; these findings verify the cutter ring performance prediction in the design stage.

## Sinusoidal variable cross-section cutter ring

### Cutter ring rock breaking mechanism

**Figure 1:**
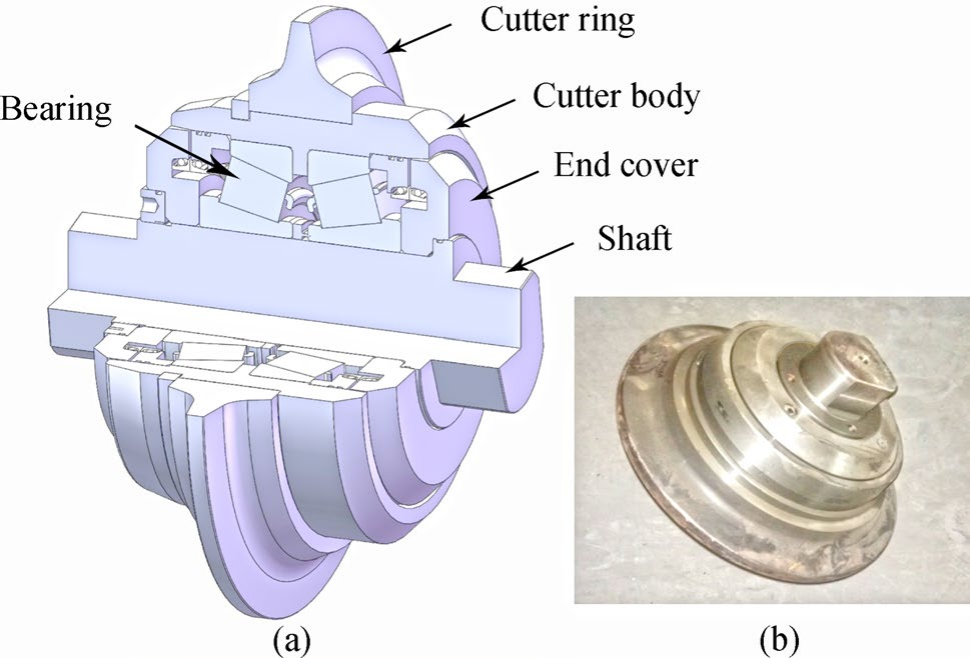
Typical disc cutter: (**a**) structure (**b**) photo.

**Figure 2:**
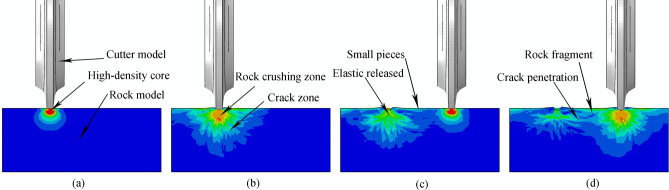
Generalized rock-breaking process: (**a**) disc cutter gradually penetrates the rock and begins to form a high density core; (**b**) cutter continues to penetrate the rock, creating cracks around the high density core, and the rock becomes partially crushed in the contact region between the rock surface and cutter; (**c**) first disc cutter leaves the rolling position, releasing elastic stress on the deep rock, adjacent disc cutter begins to penetrate and produce a second high density core; (**d**) the second cutter continues to penetrate, creating a second series of cracks around the high density core, and partially crushing the rock in the new contact region; new cracks extend to and join with previous cracks until eventually fragments become disconnected and fall off.

The disc cutter is a separate mechanical structure from the overall TBM, as shown in Fig. 1. The TBM cutter head carries multiple disc cutters for rotation and propulsion. The cutter ring blade contacts the rock first and gradually rolls through the shallow surface of the rock. The surface rock breaks and cracks under blade impact, compressive stress, tensile stress, and shear. Rock fragments form when rock cracks caused by adjacent rings are linked to each other, as shown in Fig. 2. The cutter head is continuously pushed forward, and the cutter ring rotates around both the cutter head and its own axis. Therefore, the relative motion between the disc cutter and rock mass integrates two forms of penetration and rolling motion^11,12,24,25^.

**Figure 3:**
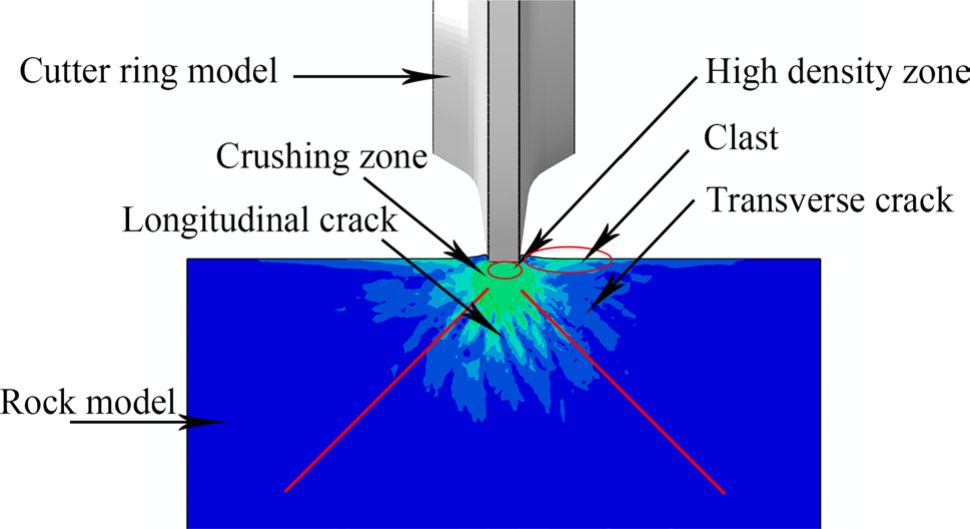
Schematic diagram of rock changes during cutter penetration.

During the process of cutting into high-strength rock, physical changes occur in the rock, including local rock becoming denser to form a high-density zone; cracks appear on either side of the blade; and there is a crushing zone below the contact surface. Cracks, including transverse cracks and longitudinal cracks, will also appear inside deeper rocks. Transverse cracks promote the detachment of fragments between cutters, while longitudinal cracks reduce the strength of rocks, as shown in Fig. 3**.**

The Colorado School of Mines (CSM) model was established from an LCM experimental database, and many previous studies have confirmed the reliability of the model^26,27^. Although the CSM model does not consider influences from rock joints, cracks, or water content under natural conditions, it remains a useful macroscopic guide for rock breakage by cutter rings under ideal conditions.

**Figure 4:**
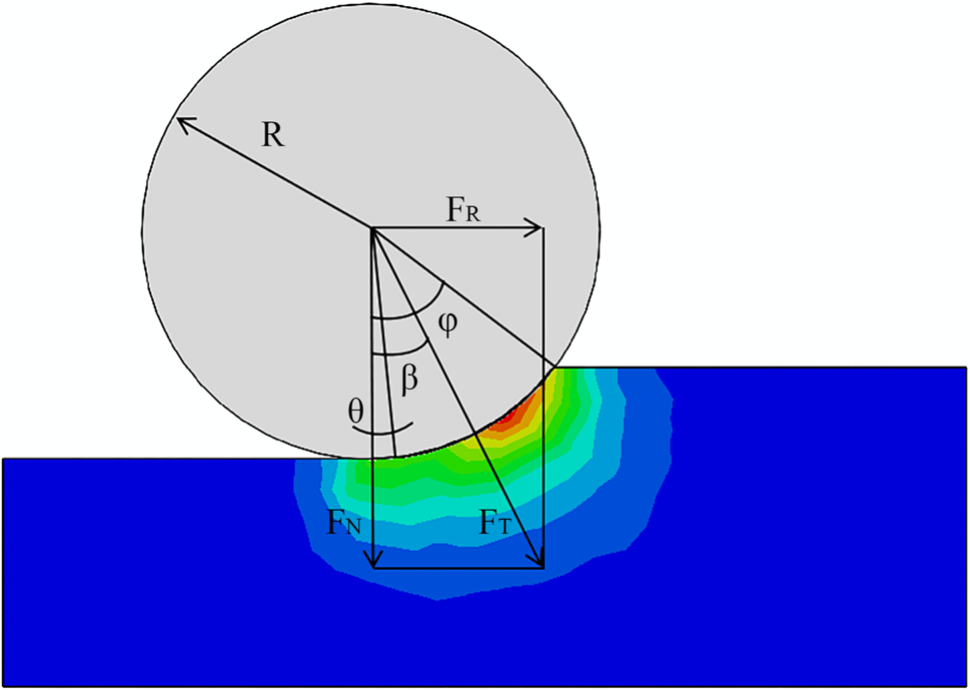
Mechanical diagram for cylindrical cutter ring.

The pressure distribution within the rock in the crushing zone for the CSM model can be expressed as

1 $${\text{P}}\left( \theta \right) = {\text{P}}_{0} \left( {\frac{\varphi }{\theta }} \right)^{\psi } ,$$

where (see Fig. 4 for the mechanical diagram) ψ is a constant pressure distribution function derived from a large number of LCM physical experimental data points, with ψ = 0.2 for a CCS cutter ring; φ is the contact angle between the rock and the tool,

2 $$\varphi = \cos^{ - 1} \left( {\frac{{{\text{R}} - {\text{p}}}}{{\text{R}}}} \right);$$

θ is the differential angle, and P_0_ is the base pressure, established by linear regression from multiple test data,

3 $${\text{P}}_{0} = {\text{C}}^{3} \sqrt {\frac{{\sigma_{{\text{c}}}^{2} \sigma_{{\text{t}}} {\text{s}}}}{{\varphi \sqrt {{\text{RT}}} }}} ,$$

where R [m] is the radius of the cutter ring, p [m] is the penetration, C is a dimensionless constant (C = 2.12), σ_c_ [MPa] is the uniaxial compressive strength of the rock, σ_t_ [MPa] is the tensile strength, s [m] is the spacing between cutters, and T [m] is the cutting edge width.

By integrating the pressure over the contact area, the total cutting force F_T_ [kN] can be expressed as

4 $${\text{F}}_{{\text{T}}} = \mathop \int \limits_{0}^{\varphi } {\text{TRP}}\left( \theta \right){\text{d}}\left( \theta \right) = \frac{{{\text{TRP}}_{0} \varphi }}{1 + \psi }.$$

The resultant force is divided into F_N_ and F_R_ [kN] in the penetration and rolling directions, i.e., perpendicular to the rock surface downward and parallel to the rock surface, respectively, along the direction of motion. This ratio is usually called the cutting factor,

5 $${\text{CC}} = \frac{{{\text{F}}_{{\text{R}}} }}{{{\text{F}}_{{\text{N}}} }} = {\text{tan }}\beta {.}$$

Assuming that the rock is isotropic with no macroscopic influence, e.g., joints, the pressure in the contact area is uniformly distributed, and |β|= φ/2. Therefore,

6 $${\text{F}}_{{\text{T}}} = {\text{F}}_{{\text{R}}} \tan^{ - 1} {\text{CC}}{.}$$

The above formula shows that the influence of the blade width T on the resultant force F_T_ of the cutter is direct; that is, if the blade width changes, the resultant F_T_ will also change.

### Design of the sinusoidal VCS cutter ring

**Figure 5:**
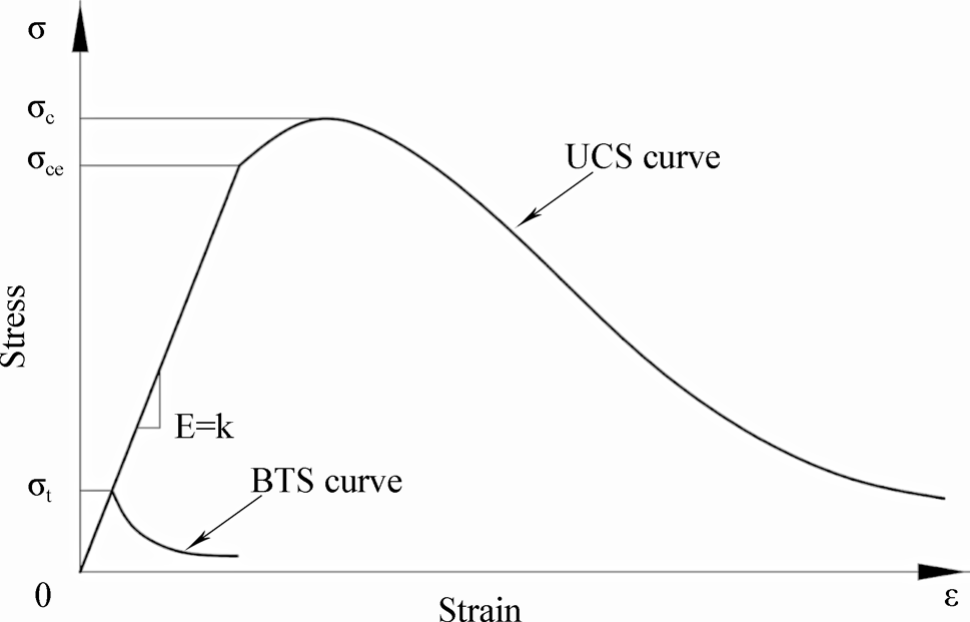
Diagram of stress strain curves of general rock materials.

Rock is a brittle material with a compressive strength much greater than the tensile strength^28^, as shown in Fig. 5. Thus, for most engineering uses, σ_c_≈10*σ_t_.

**Figure 6:**
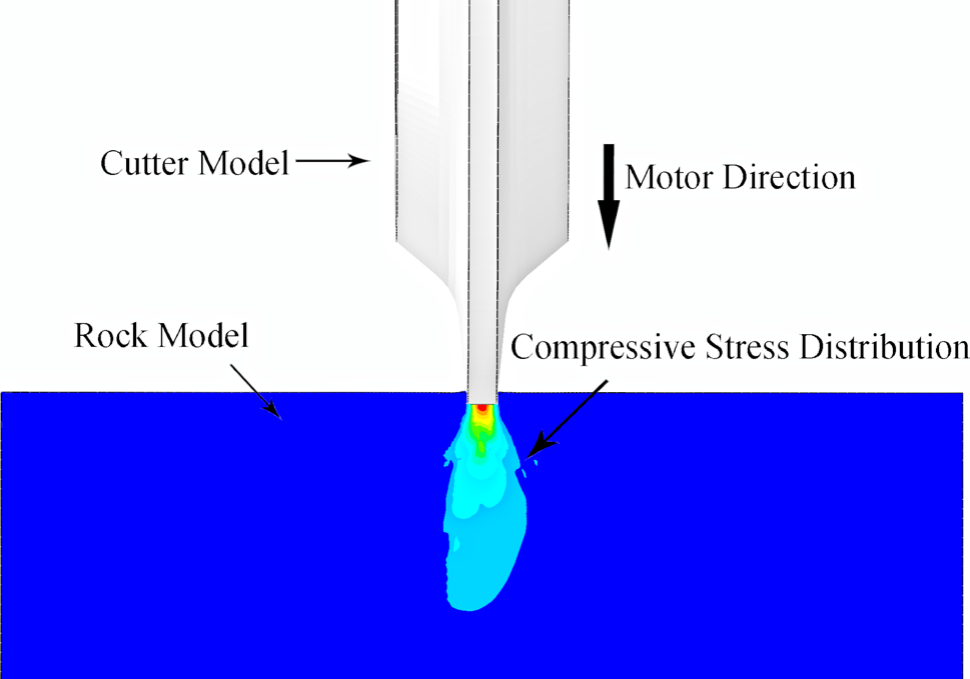
The compressive stress on the rock when the cutter ring is pressed into the rock.

**Figure 7:**
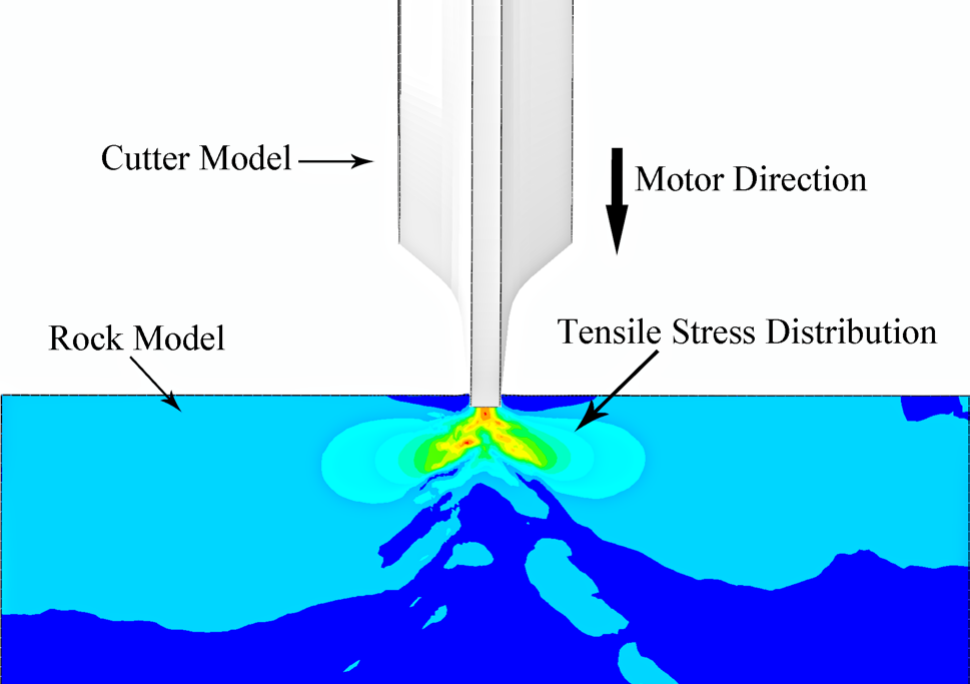
The tensile stress on the rock when the cutter ring is pressed into the rock.

Previous studies simulating the cutter ring being pressed into rock showed compressive and tensile stress ranges of action, as shown in Figs. 6 and 7. The compressive stress is concentrated in the normal direction to the contact surface, and the side inclination of the cutter ring in the direction of tensile stress is related to the width of the blade^29,30^. Under these contact conditions, the tensile stress in the rock is less than the compressive stress.

Tunnel boring machines often encounter rock masses with good integrity during mountain tunnel construction, which generally follows the continuity, homogeneity, and isotropy hypotheses. Thus, tensile stress better damages the rock under equal tensile and compressive stress conditions, and a better crushing effect can be obtained by increasing the tensile stress from the cutter ring.

**Figure 8:**
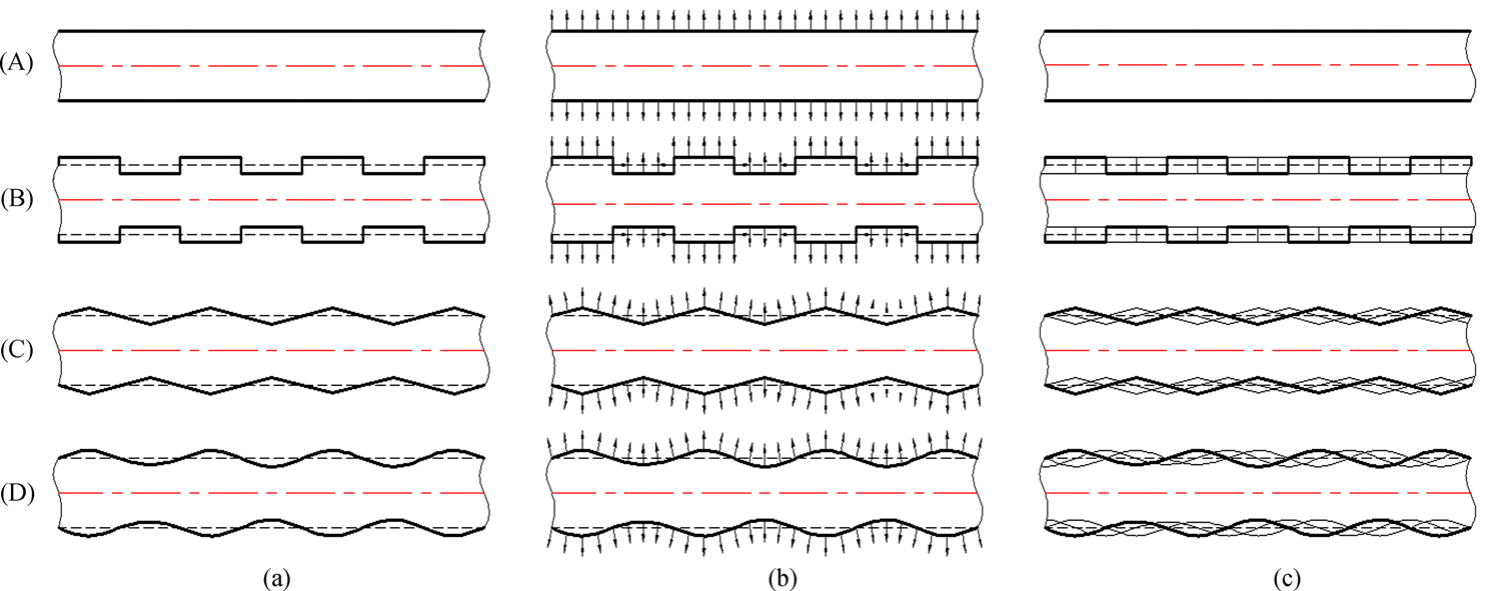
Cutter ring blade rolling path: (**A**) CCS cutter ring, (**B**) square VCS cutter ring; (**C**) triangular VCS cutter ring; and (**D**) sinusoidal VCS cutter ring; Column (**a**) single rolling track, (**b**) tangential force on the rock generated by cutter rings rolling, and (**c**) multiple rolling paths for the cutter rings.

Therefore, various changes to the CCS cutter ring blade part may have different effects, as shown in Fig. 8. In column (a), the track of rolling one time for various cutter rings is shown, and their contact areas with the rock are equal. In column (b), the force direction of the blade side of each cutter ring is shown. The force direction of the CCS cutter ring is relatively simple, while the force direction of the sinusoidal VCS cutter ring is complex. In column (c), the traces of various cutter rings rolling the same path many times are shown. The rolling trace of the VCS cutter rings is wider than that of the CCS cutter rings. Replacing a CCS cutter ring with a VCS cutter ring could help the cutter ring generate more tensile stress on the rock, promoting rock breakage. However, the square and triangular cutter ring edges increase the impact on the ring and hence are unfavorable for production and service life. On the other hand, the sinusoidal VCS cutter ring ensures that the force direction on the blade is more uniform and smoothly changing. Thus, the sinusoidal VCS ring has better engineering adaptability and performance than the square and triangular profile rings.

**Figure 9:**
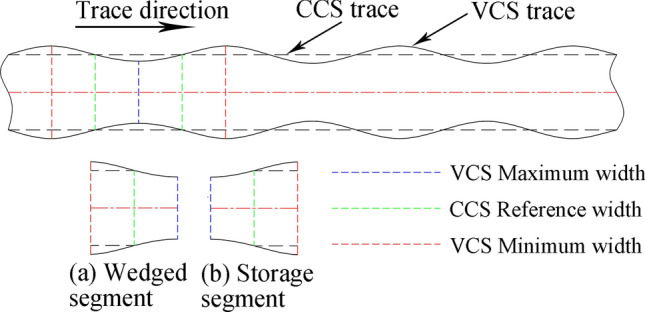
Segmentation diagram of VCS cutter ring rolling track.

**Figure 10:**
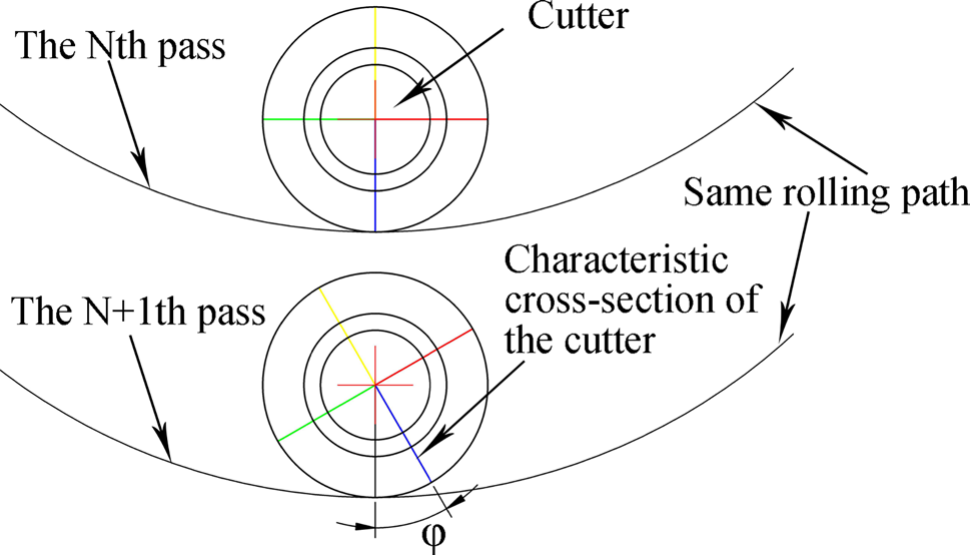
Dislocation Angle diagram.

**Figure 11:**
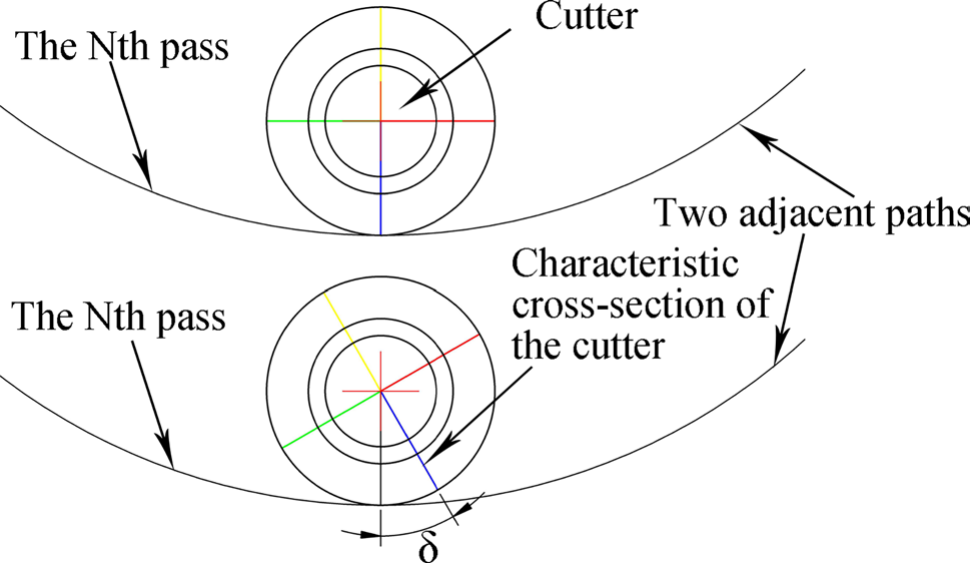
Adjacency Angle diagram.

**Figure 12:**
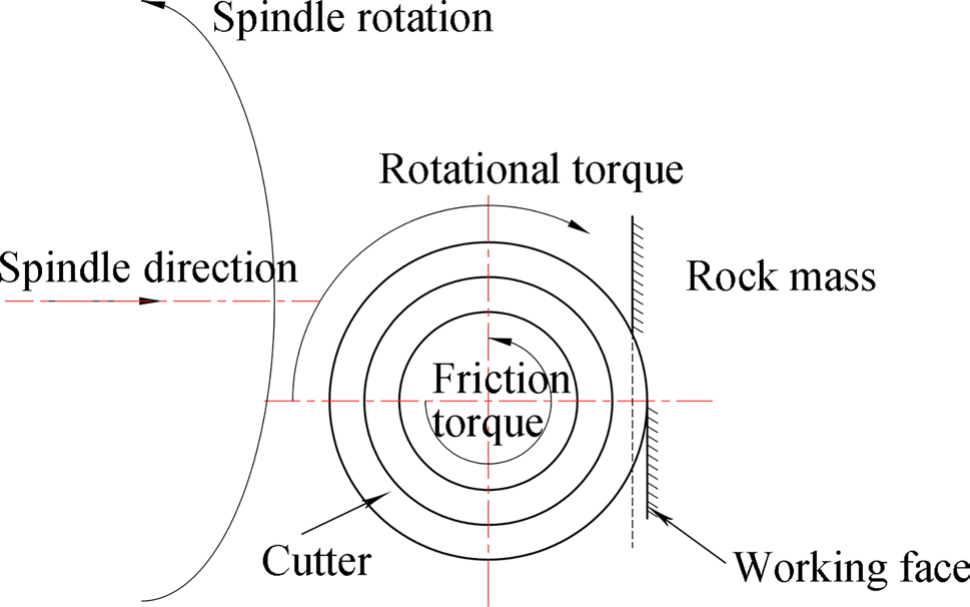
Cutter ring torque diagram.

**Figure 13:**
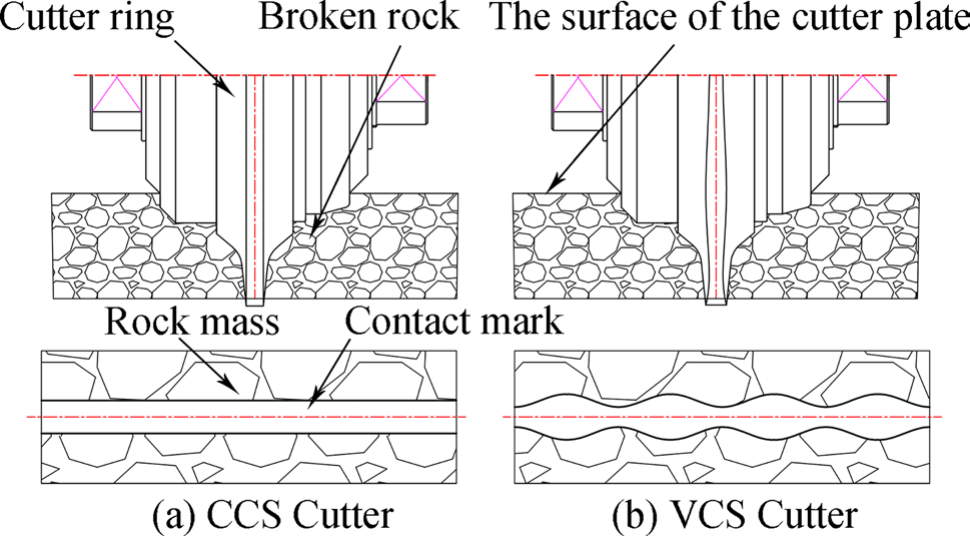
Frictional environment comparison diagram.

Compared with the CCS cutter ring, the VCS cutter ring has the following differences in terms of its rock breaking mechanism and performance:

1. The contact track of the VCS cutter ring changes periodically, and each cycle includes two segments, namely, the wedge segment and the energy storage segment, as shown in Fig. 9. When the contact between the cutter ring and the rock is in the wedge segment, the contact area gradually increases, and the thrust applied by the rock promotes the extension of transverse cracks in the rock. When the contact position is in the energy storage segment, the interaction force gradually decreases in preparation for the next wedge.
2. The VCS cutter rolls in its path, and the contact point of the tool ring is different every time the tool ring passes through the same position; this contact point is defined as the dislocation angle φ, as shown in Fig. 10. The dislocation angle increases the damage width on the path, as shown in column (c) of row (D) of Fig. 8.
3. In addition, when two adjacent VCS cutter rings pass through the same radius line and when the two paths are focused on the rock, there will be an angle between the same characteristic cross section, which is defined as the adjacency angle δ, as shown in Fig. 11. Under the combined action of the adjacency angle and the dislocation angle, the rocks between the adjacent VCS cutter ring rolling paths are subjected to fluctuating loads, which promote rock cracking and fall.
4. The rolling torque of the cutter ring comes from the frictional force of the rock, as shown in Fig. 12. When the torque generated by the frictional force is greater than the starting torque of the disc cutter, the cutter rotates. In many cases, the partial wear failure of the cutter ring is caused by a friction torque that is too small. Compared with the CCS cutter ring, the VCS cutter ring is subjected to greater friction, including the friction generated by the free gravel between the rock surface and the cutter plate and the friction between the cutter blade and the rock, as shown in Fig. 13.

As discussed above, sinusoidal VCS cutter rings produce more uniform waves during rock failure, which promotes rock crack formation and extension. A sinusoidal VCS cutter ring will reduce energy loss, and increasing crack extension will increase surface rock loss, increasing the damage volume. Therefore, it is likely that the sinusoidal VCS cutter rings will require less energy and lower specific energy to destroy the same volume of rock as the other designs, improving the TBM working efficiency. An FEM simulation was used to verify this effect.

**Figure 14:**
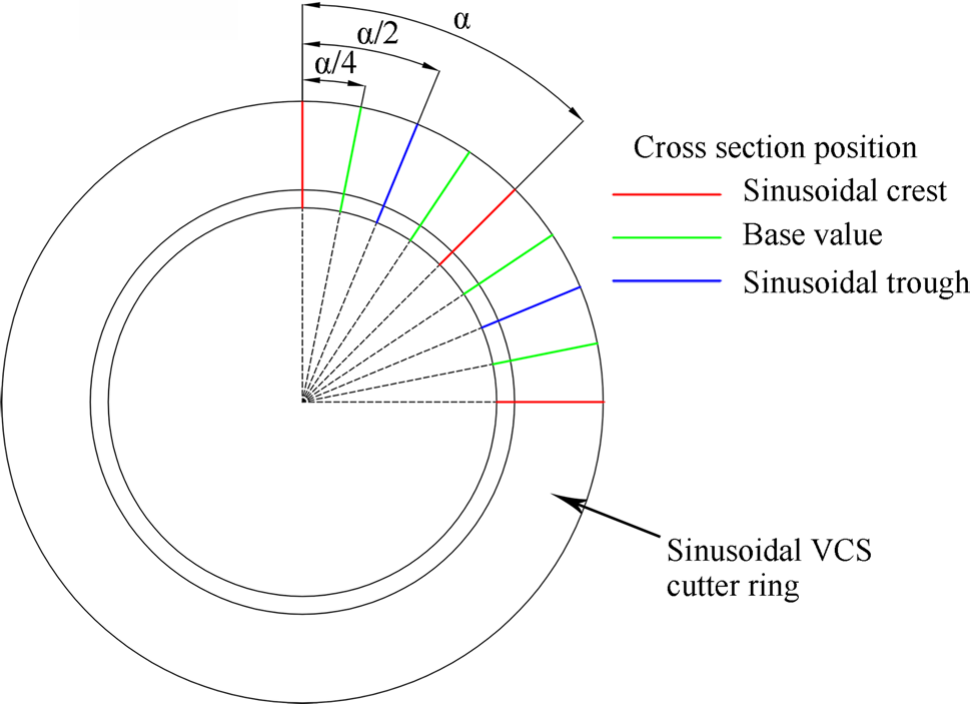
Number of cross section changes for the sinusoidal VCS cutter ring; α corresponds to a complete waveform on the blade.

The cutter ring blade must be redesigned to realize the motion path for the sinusoidal VCS cutter ring. The unique design features include the cross section change amplitude, A, and the number of cross section changes per circle, N, compared with those of CCS cutter rings. Other design parameters follow CCS cutter ring characteristics, including cutter ring diameter, D, blade width T, and blade angle γ. The cross-sectional change amplitude can be expressed as

7 $${\text{A}} = \frac{{{\text{T}}_{{{\text{max}}}} + {\text{T}}_{{{\text{min}}}} }}{2},$$

where T_max_ and T_min_ are the maximum and minimum blade widths, respectively, for the section and the number of cross sections changes per circle as follows:

8 $${\text{N}} = \frac{360^\circ }{{\upalpha }},$$

where α is the angle corresponding to a complete waveform on the blade, as shown in Fig. 14. Considering the cutter ring dynamic balance, N should be an integer greater than 1.

**Figure 15:**
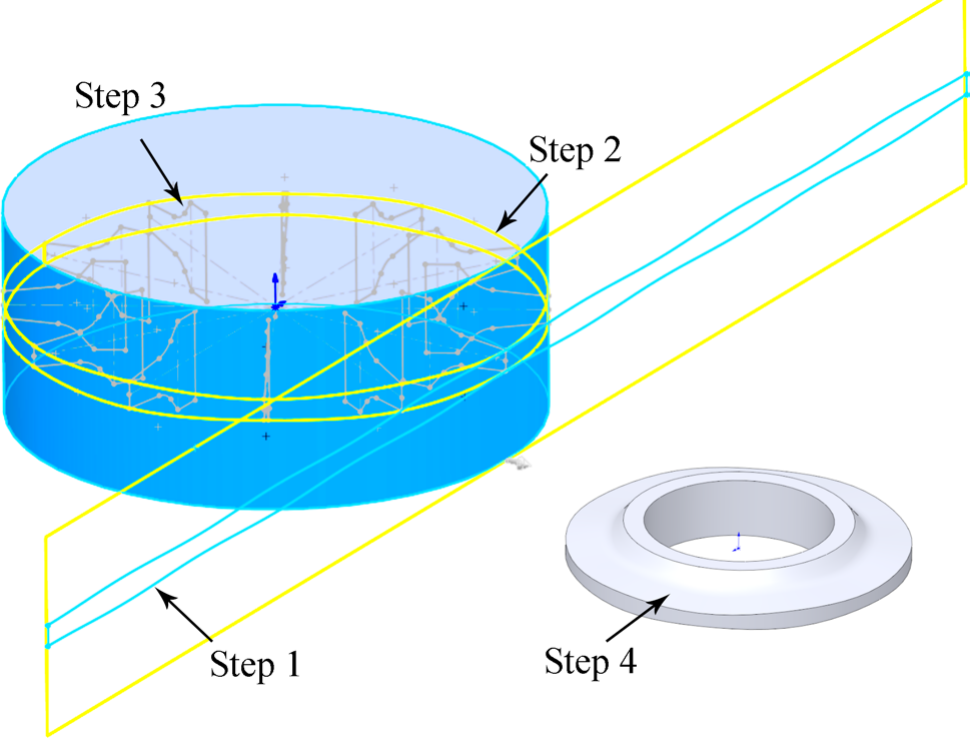
Drawing method for sinusoidal VCS cutter ring 3D model.

The drawing method for the digital sinusoidal VCS cutter ring model is shown in Fig. 15.

*Step 1* The blade trace curve is drawn on the plane where the blade tangent line is located.

9 $${\text{Y}} = \frac{{\text{A}}}{2}\sin \left( {{\omega X} - \frac{{\uppi }}{2}} \right) + {\text{K X}} \in \left( { - {\uppi },{\uppi }} \right),$$

where

10 $${\text{K}} = \frac{{\text{T}}}{2}$$

is the base value for the curve; and

11 $${\upomega } = \frac{{2{\uppi }}}{{\text{t}}}$$

is the cross-sectional change frequency, and

12 $${\text{t}} = \frac{{2{\pi R}}}{{\text{N}}}$$

is the change period.

Thus,

13 $${\text{Y}} = \frac{{\text{A}}}{2}\sin \left( {\frac{{\text{N}}}{{\text{R}}}{\text{X}} \mp \frac{{\uppi }}{2}} \right) \pm \frac{{\text{T}}}{2}{\text{ X}} \in \left( { - {\uppi },{\uppi }} \right).$$

*Step 2* wraps the curve on the surface of the cylinder where the blade is, forming a ring to close the curve.

*Step 3* is to scan each section outline along the ring curve in turn.

*Step 4* is to establish the solid model after sweeping.

This paper sets N = 2, 3, 4, 5, and 6 and A = 1, 1.5, 2, 2.5, and 3 mm for orthogonal combination to establish a sinusoidal VCS cutter ring model library for future use.

## Simulation modeling and verification

### Rock-like material model and validation

Rock material properties include strength, stiffness, brittleness, fracture toughness, elastic modulus, compressibility, mineral composition, porosity, water absorption, density, and Poisson's ratio. It is difficult to define all the properties of an FEM; hence, we usually define the key properties, i.e., strength, density and elastic modulus, and create assumptions regarding uniformity, continuity and isotropy. Thus, the defined materials are similar only to real rock materials, i.e., rock-like materials. Many previous studies have verified the similarity between simulated rock-like materials and real rock using the FEM, and the main factors affecting rock breakage are compressive strength and tensile strength^31–34^.

The influence of various factors on the material strength was investigated using a group of materials with the same density, Poisson's ratio, and elastic modulus but different uniaxial compressive and tensile strengths. A rock-like material library was established by combining the concrete damage constitutive model with the strength and damage coupling from Abaqus software and the orthogonal test method. The main design parameters are shown in Table 1.

**Table 1:** Model properties of rock-like materials.

Parameter	Uniaxial compressive strength, σ [MPa]	Tensile strength, τ [MPa]	Elasticity Modulus, E [GPa]	Density, Ρ [kg/m^3^]	Poisson's ratio, μ
Value	60, 90, 120, 150, 180	3, 4, 5, 6, 7	60	2650	0.23

Abaqus provides a constitutive model for calculating rock tensile and compressive strength,

14 $$\upsigma_{{\text{t}}} = \upsigma_{{\text{t}}} \left( {\upvarepsilon_{{{\text{tp}}}} ,\;\upvarepsilon_{{{\text{tr}}}} } \right)$$

and

15 $$\upsigma_{{\text{c}}} = \upsigma_{{\text{c}}} \left( {\upvarepsilon_{{{\text{cp}}}} ,\;\upvarepsilon_{{{\text{cr}}}} } \right),$$

where subscripts t and c refer to tension and compression, ε_tp_ and ε_cp_ are the equivalent plastic strains, and ε_tr_ and ε_cr_ are the equivalent plastic strain rates.

The unloading response is weakened whenever a rock specimen is unloaded from any point on the strain-softening branch of the stress‒strain curves, i.e., when the material elastic stiffness is damaged (or degraded). The elastic stiffness degradation is characterized by damage variables d_t_ and d_c_, which are assumed to be functions of the plastic strains, i.e.,

16 $${\text{d}}_{{\text{t}}} = {\text{d}}_{{\text{t}}} \left( {{\upvarepsilon }_{{{\text{tp}}}} } \right);{ }0 \le {\text{d}}_{{\text{t}}} \le 1,$$

and

17 $${\text{d}}_{{\text{c}}} = {\text{d}}_{{\text{c}}} \left( {{\upvarepsilon }_{{{\text{cp}}}} } \right);{ }0 \le {\text{d}}_{{\text{c}}} \le 1;$$

where d_t_, d_c_ = 0 represents undamaged material and d_t_, d_c_ = 1 represents total loss of strength.

Let E_0_ be the initial (undamaged) elastic stiffness of the material; then, the stress‒strain relations under uniaxial tension and compression loading are

18 $${\upsigma }_{{\text{t}}} = \left( {1 - {\text{d}}_{{\text{t}}} } \right){\text{E}}_{0} \left( {{\upvarepsilon }_{{\text{t}}} - {\upvarepsilon }_{{{\text{tp}}}} } \right),$$

and

19 $${\upsigma }_{{\text{c}}} = \left( {1 - {\text{d}}_{{\text{c}}} } \right){\text{E}}_{0} \left( {{\upvarepsilon }_{{\text{c}}} - {\upvarepsilon }_{{{\text{cp}}}} } \right);$$

and define effective tensile and compressive cohesion stresses as

20 $${\upsigma }_{{{\text{te}}}} = \frac{{{\upsigma }_{{\text{t}}} }}{{\left( {1 - {\text{d}}_{{\text{t}}} } \right)}} = {\text{E}}_{0} \left( {{\upvarepsilon }_{{\text{t}}} - {\upvarepsilon }_{{{\text{tp}}}} } \right)$$

and

21 $${\upsigma }_{{{\text{ce}}}} = \frac{{{\upsigma }_{{\text{c}}} }}{{\left( {1 - {\text{d}}_{{\text{c}}} } \right)}} = {\text{E}}_{0} \left( {{\upvarepsilon }_{{\text{c}}} - {\upvarepsilon }_{{{\text{cp}}}} } \right),$$

**Figure 16:**
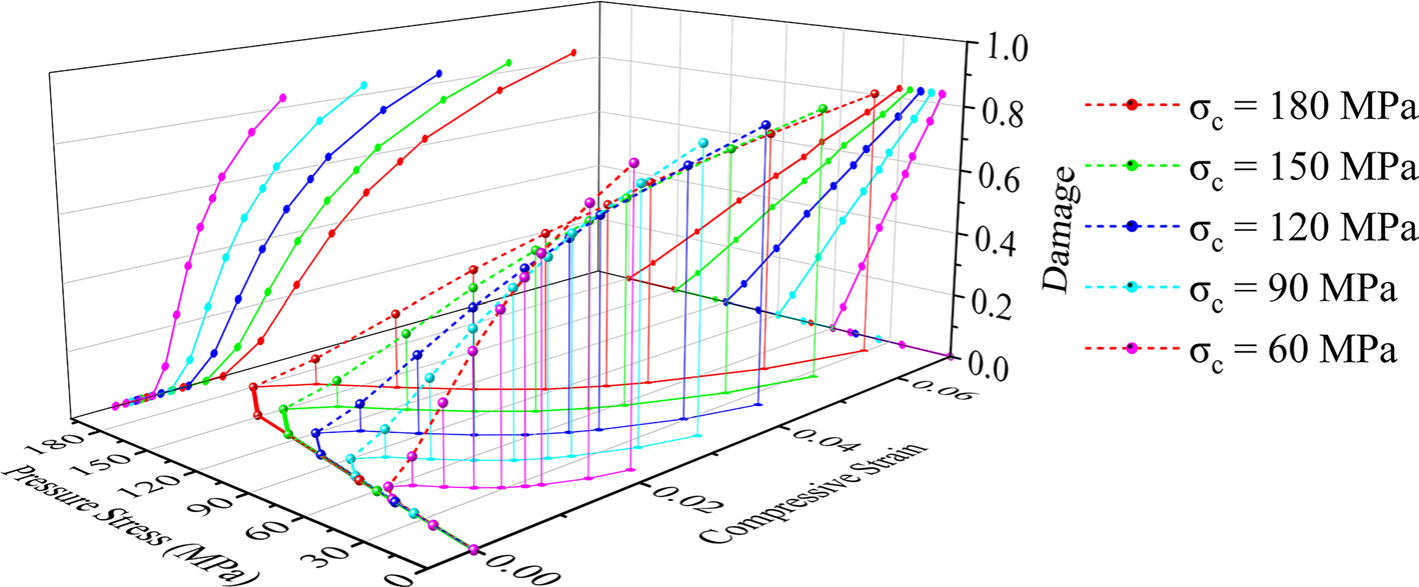
Compressive stress‒strain-damage constitutive relationships for a rock-like model.

**Figure 17:**
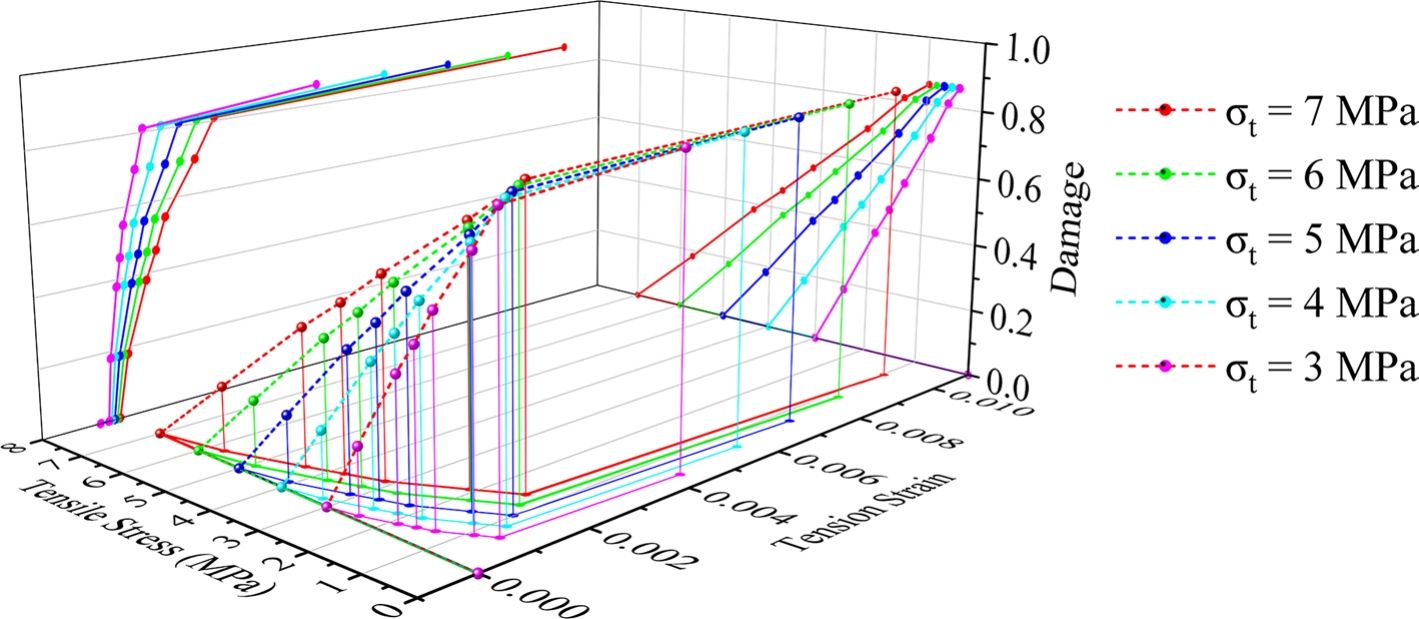
Tensile stress‒strain-damage constitutive relationships for a rock-like model.

Moreover, Figs. 16 and 17 show the functional relationship curves for stress, strain and damage, respectively, to ensure that the rock damage simulation is controllable and comparable.

**Figure 18:**
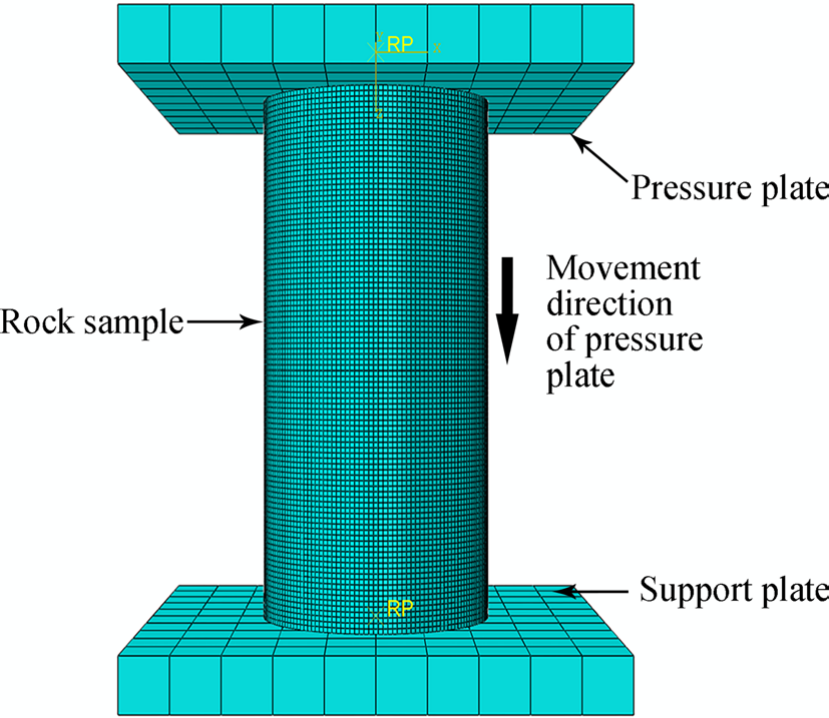
UCS simulation test assembly for rock.

**Figure 19:**
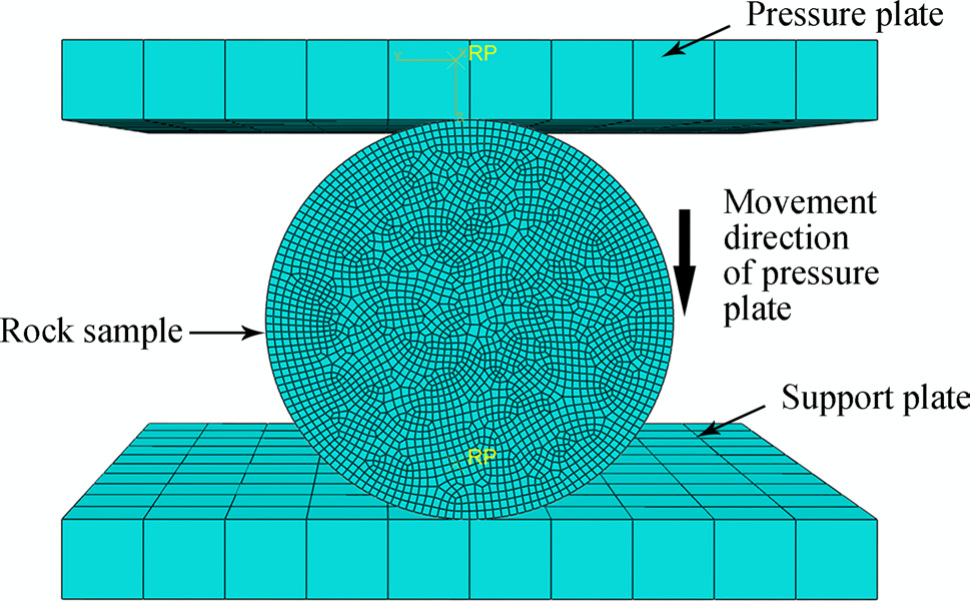
BTS simulation test assembly for rock.

To verify the effectiveness of the rock-like materials, uniaxial compression strength (UCS) and Brazilian tensile strength (BTS) tests were performed on material models in the material library. Figures 18 and 19 show the UCS and BTS simulation assemblies, respectively, comprising two identical cuboids 100 × 100 × 10 mm (length, width, height) as discrete rigid bodies to serve as support and pressure plates.

In the load and boundary condition setting module for the two simulation models, the 6 degrees of freedom for the support plate are completely fixed, and the pressure plate can only move uniformly in a straight line along the opposite Z axis direction at a single point with a speed of 1 mm/s. Movement times for pressure plates = 5 and 3 s for the UCS and BTS models, respectively. The rock sample was divided into hexahedra with a 0.8 mm side length, and the support and pressure plates were divided into hexahedra with a 10 mm side length. The simulation stress‒strain and damage data can then be compared with the experimental results.

According to the mechanical parameters of the rock-like materials, 25 groups of orthogonal tests were designed (Tables 1 and 2), and UCS and BTS simulations were performed on the models.

**Table 2:** Rock-like material parameter values.

No	1	2	3	4	5	6	7	8	9	10	11	12	13
σ_c_ [MPa]	60	180	60	90	60	150	180	150	90	120	120	60	150
σ_t_ [MPa]	5	5	3	7	6	5	6	3	5	7	5	7	7

No	14	15	16	17	18	19	20	21	22	23	24	25	
σ_c_ [MPa]	180	180	150	90	180	120	60	150	120	120	90	90	
σ_t_ [MPa]	7	4	4	4	3	6	4	6	4	3	3	6	

**Figure 20:**
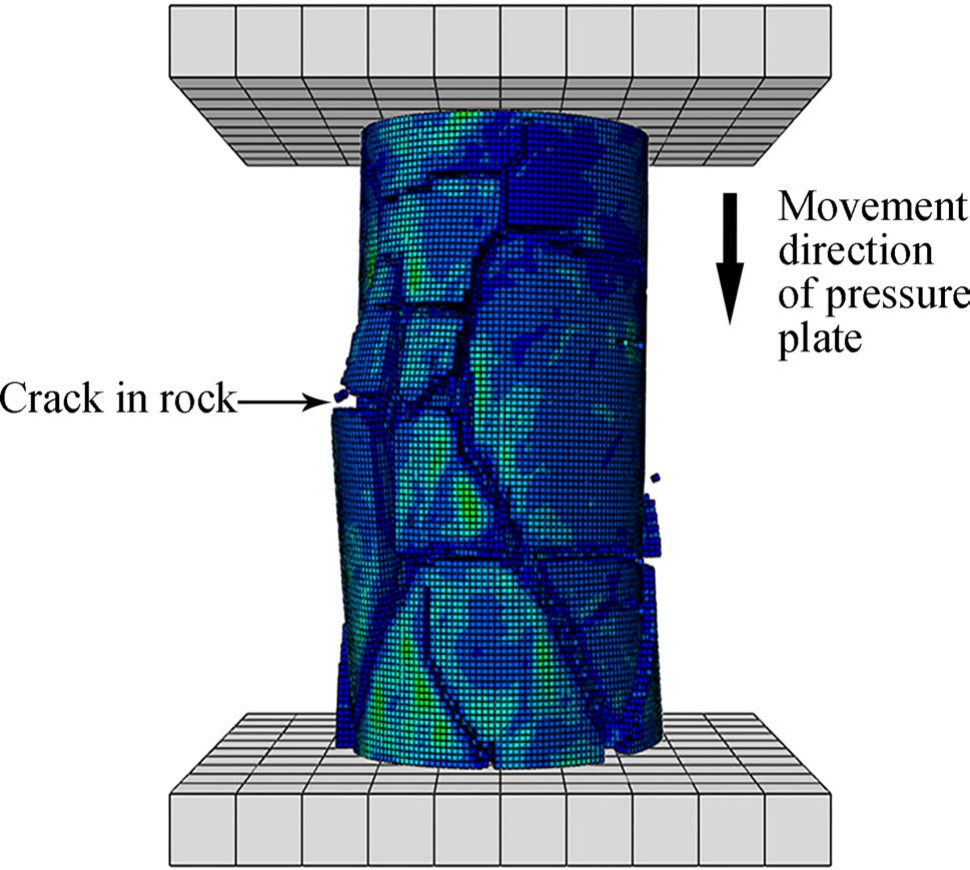
UCS simulation results for rock samples.

**Figure 21:**
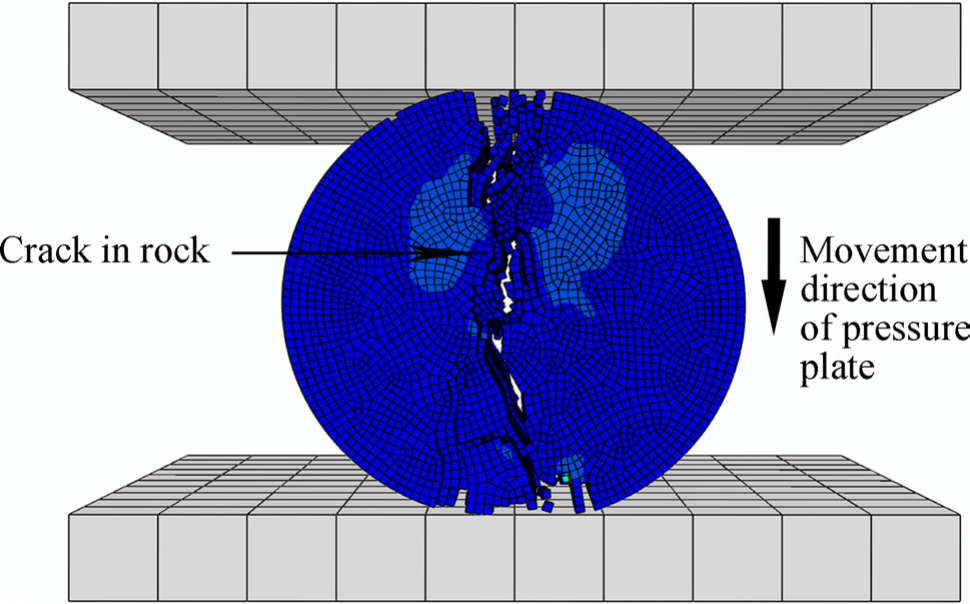
BTS simulation results for rock samples.

Figures 20 and 21 show the UCS and BTS simulation outcomes, respectively, for one of the 25 simulation tests. Comparing the damage distribution and fracture location for the rock model against those of actual samples confirms that the simulation results are consistent with real outcomes.

**Figure 22:**
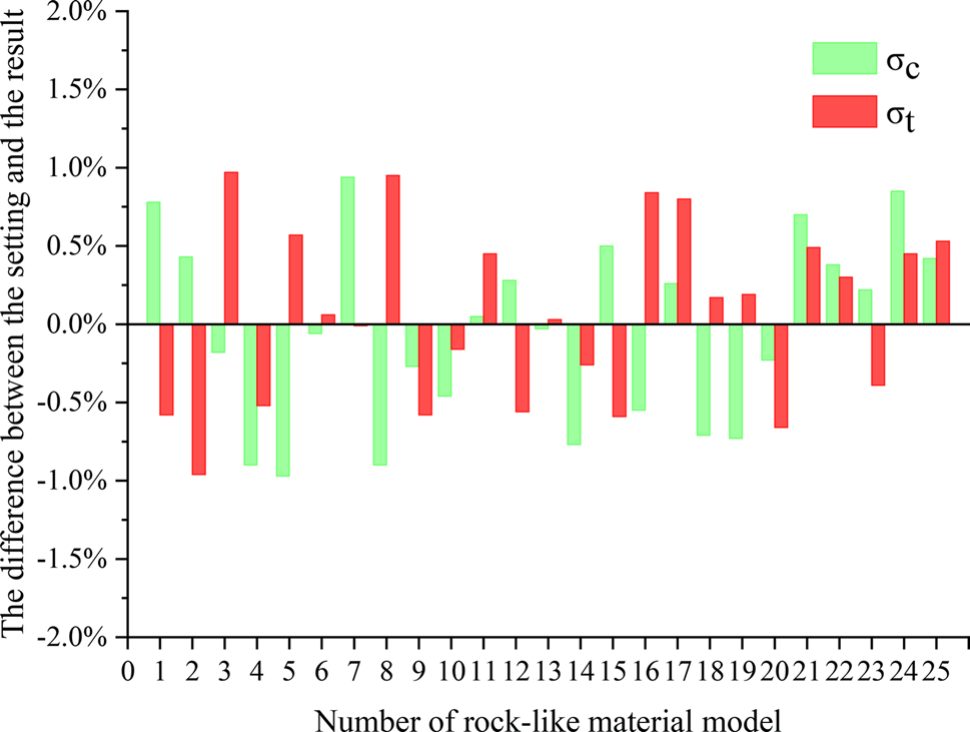
Strength parameters for 25 rock-like material models compared with simulated mechanical experiments.

Figure 22 compares the average strength curves for all the rock-like material elements obtained via simulation against the real outcomes. The deviation between the simulated and actual rock-like material strengths is always less than 1%. This finding verifies the controllability and comparability of this rock-like material model and validates the material setting method.

In conclusion, the method of establishing a simulation model of rock-like materials adopted in this paper is effective.

### Linear cutting machine modeling and validation

#### Penetration simulation

**Figure 23:**
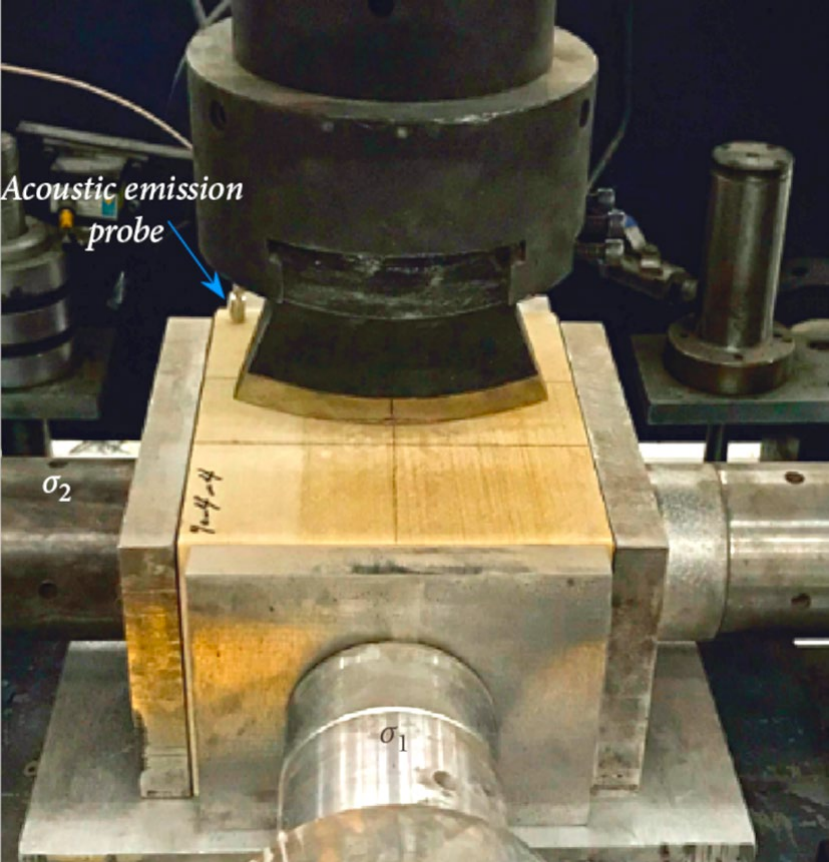
Penetration force test equipment (Ying ji^37^).

The penetration force is an important index for studying hob performance, and many scholars have used experimental equipment to test the rock breaking force^35–37^, as shown in Fig. 23. In this paper, a simulation model of cutter penetration into rock is established via FEM to test the force of cutter penetration into rock.

**Figure 24:**
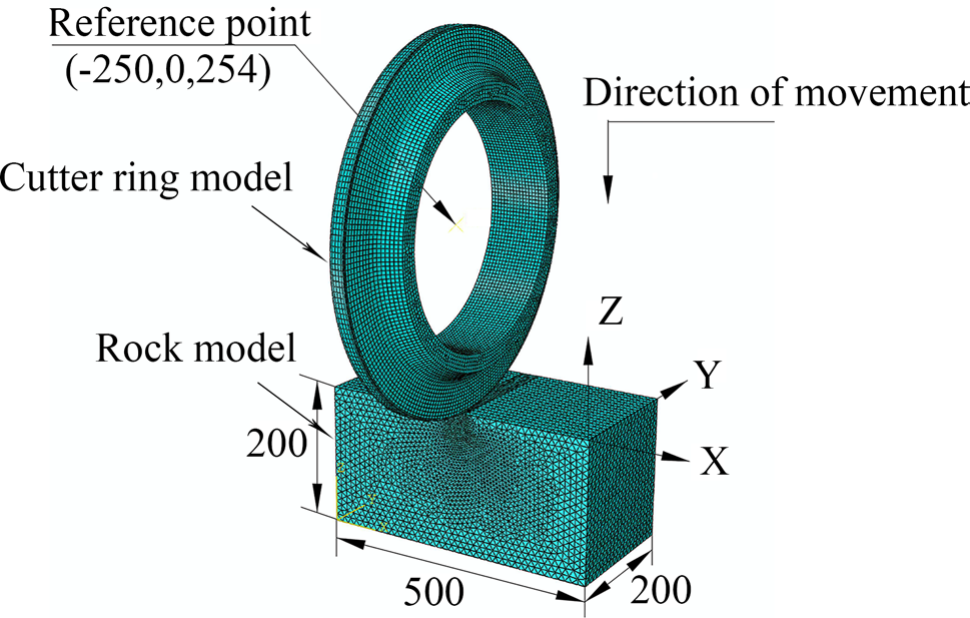
Simulation model of cutter penetration into rock.

The modeling process included the following steps:

1. Modeling a generic 20-inch CCS cutter ring with diameter = 508 mm, blade angle = 20°, and 5 possible blade widths = 16, 18, 20, 22, and 24 mm. The material for each cutter ring was set as a discrete rigid body, regardless of wear and deformation. The rock-like material model was a cuboid 500 × 200 × 200 mm (length, width, thickness). The rock-like material was set as described in Section Rock-like material model and validation. To simplify the experimental process, the ratio of the compressive strength to the tensile strength of the material was determined to be 10, and rock-like material models with compressive strengths of 60 MPa, 80 MPa, 100 MPa, 120 MPa and 140 MPa were established.
2. The model was assembled as shown in Fig. 24. The cutter ring model is tangent to the upper surface of the rock-like material model, and the cutting point is located on the edge line of the rock-like model.
3. The analysis step was set to display the dynamic mode for 5 s to test the target selection force and displacement.
4. The mesh of the cutter ring model is a hexahedron with a side length of 10 mm, and the mesh of the rock-like material is a tetrahedron with a side length of 4 mm.
5. In the boundary conditions module, the lower surface of the rock-like model is set to be fixed, the surrounding surface is set to be symmetrical, and the upper surface is not constrained. The velocity of the cutter ring model along the Z negative direction is 1 mm/s, and the other degrees of freedom are set to 0.

**Figure 25:**
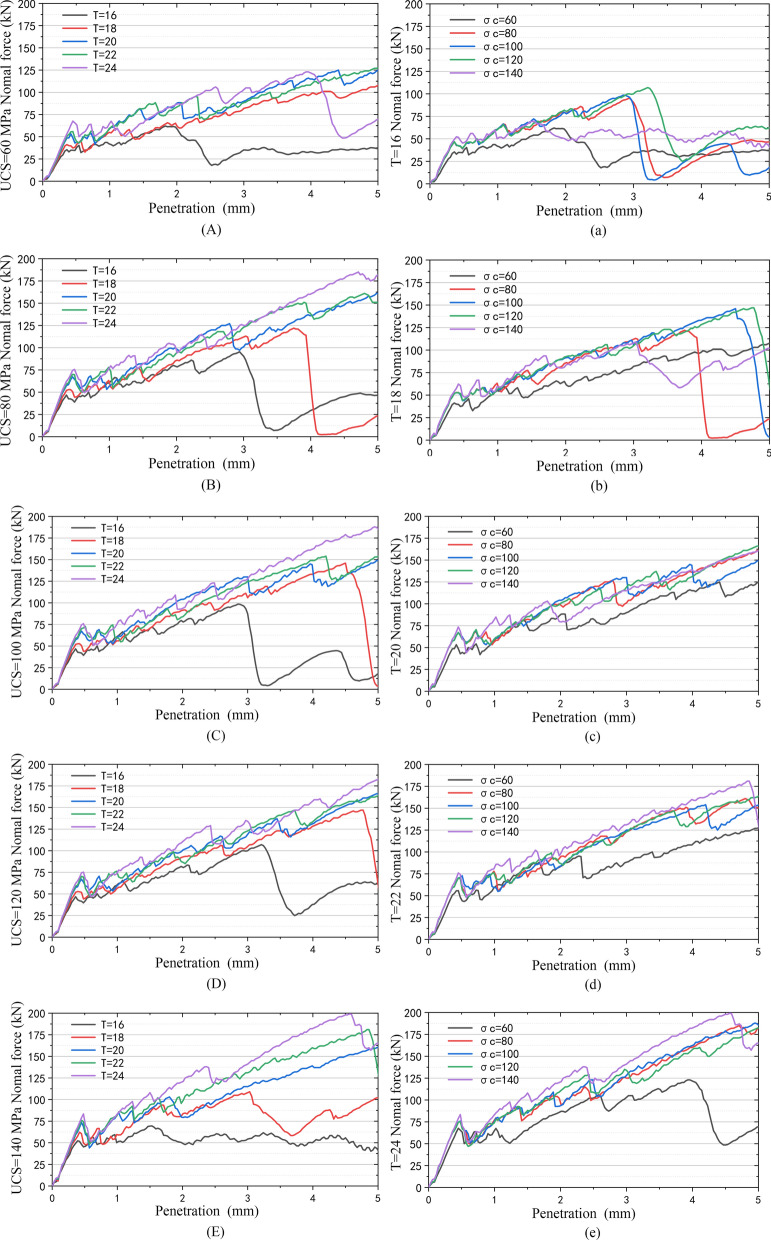
Penetration force curve: (**A**), (**B**), (**C**), (**D**) and (**E**) are penetration force curves of the same rock with different blade widths; (**a**), (**b**), (**c**), (**d**) and (**e**) are penetration forces of the same cutter on rocks with different strengths.

The test results are shown in Fig. 25.

The results of the simulation test are similar to those of other scholars using the testing machine. The penetration force increases with increasing rock strength and blade width, which further proves that the establishment of a simulation model for such rock-like materials is effective.

#### Single-cutter simulation

**Figure 26:**
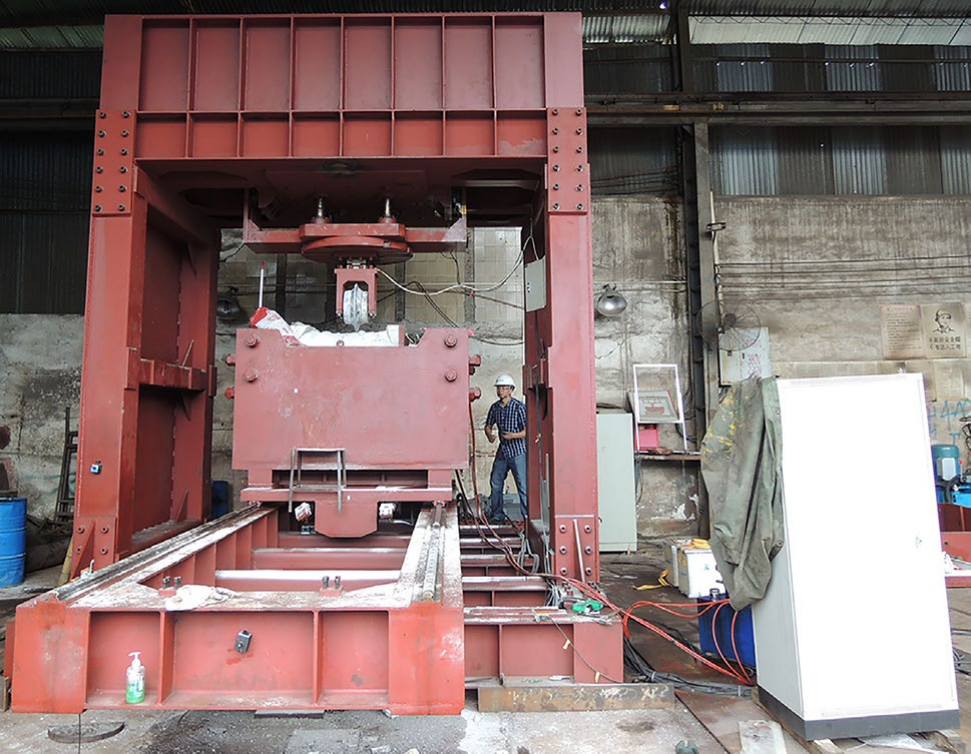
Full-size LCM from University of Science and Technology, Beijing^38^.

The simulation model is designed according to the working principle of the full-size LCM in the literature records, as shown in Fig. 26^38^. The purpose was to test the force conditions for 25 rock-like material models rolled by a cutter ring with the same outer diameter and different blade widths and compare the simulation results with the calculated results for the CSM model.

**Figure 27:**
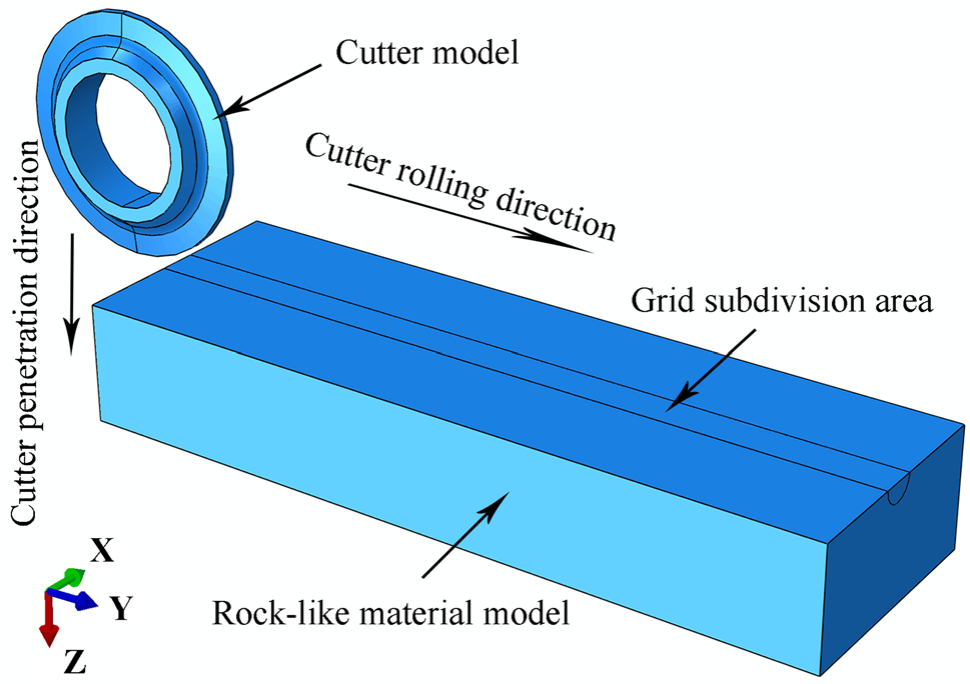
Single-cutter rolling simulation diagram.

The modeling process included the following steps:

1. Modeling a generic 20-inch CCS cutter ring with diameter = 508 mm, blade angle = 20°, and 5 possible blade widths = 16, 18, 20, 22, and 24 mm. The material for each cutter ring was set as a discrete rigid body, regardless of wear and deformation. The rock-like material model was a cuboid 1800 × 500 × 250 mm (length, width, thickness) to ensure that the cutter could roll a full circle on the rock. The rock-like material was set as described in Section Rock-like material model and validation, with 25 materials. The upper surface of the rock-like material was subdivided into a grid around an initial central 20 mm radius circle.
2. The model was assembled as shown in Fig. 27. The axial symmetry plane for the cutter ring coincides with the rock axial center plane. The cutter ring model was tangent to the rock model upper surface, and the cutting point was 100 mm from the rock material.
3. The analysis step was set as dynamic display analysis.
  - Penetration. With the cutter ring model motion along the Z axis (Fig. 27), the depth was set to 2, 3, 4, 5, or 6 mm, corresponding to the motion speed setting, and the time was set to 1 s.
  - Rolling. The rock-like motion of the cutter ring model was fixed along the Y axis (Fig. 27), the time was 40 s, and the number of samples was 200.
4. Meshes. The meshes are divided, and the rock-like model contact surfaces are set. The meshes were set as tetrahedra with 1 mm and 3 mm sides for the subdivision area and other meshes, respectively. The contact mode is set to surface to surface type, where the active surface is the cutter ring model outer surface and the passive surface is the rock model surface.
5. Model constraints. The bottom surface of the rock-like model is a fixed constraint, all four sides are symmetrical constraints, and the upper surface is constraint free. The cutter ring model motion direction and speed were set.
  c. The cutter ring model forward moving speed along the Z axis was 2, 3, 4, 5, or 6 mm with all the other degrees of freedom closed.
  d. The cutter ring model moves along the Y axis and rotates around the X axis. The moving and rotating speeds are 50.8 mm/s and 0.2 rad/s, respectively.

**Figure 28:**
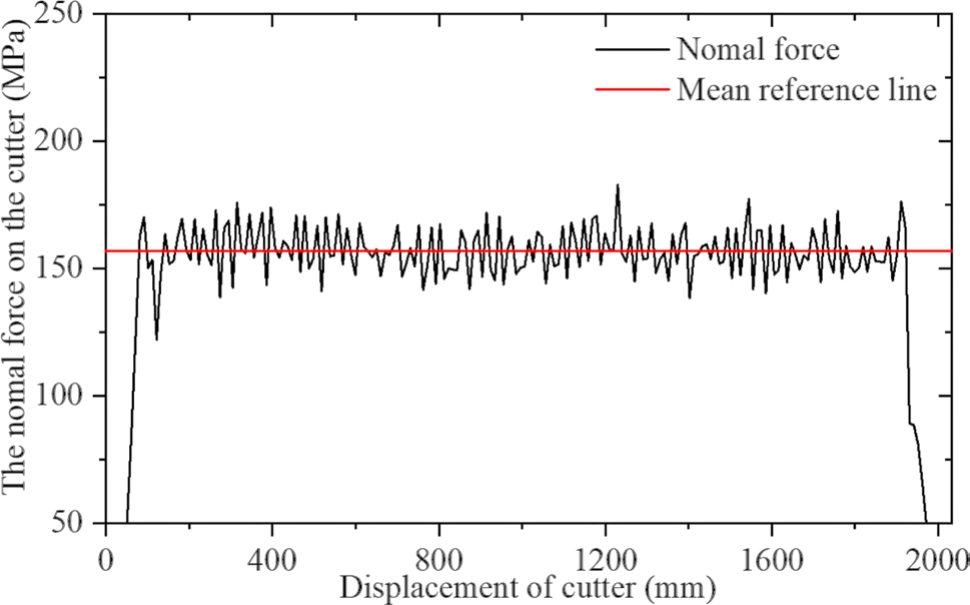
Simulation results for CCS single-cutter rolling, normal force‒displacement curve.

**Figure 29:**
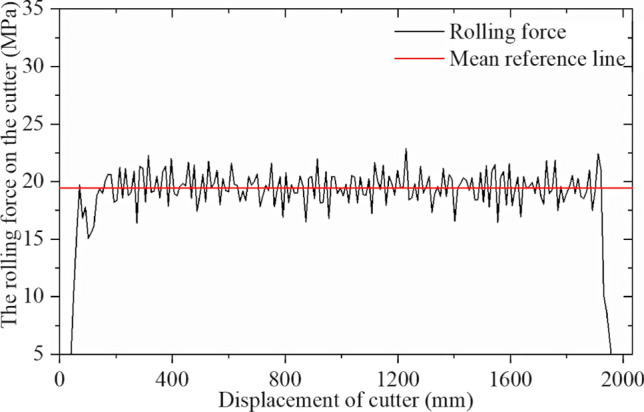
Simulation results for CCS single-cutter rolling, rolling force‒displacement curve.

Table 3 shows the orthogonal test table for the modeling as described. Simulations were performed in sequence, and the cutter ring positive and rolling force were extracted. Figures 28 and 29 show simulation result 1.

**Table 3:** Orthogonal test set for CCS cutter simulations.

No	1	2	3	4	5	6	7	8	9	10	11	12	13
σ_c_ [MPa]	60	180	60	90	60	150	180	150	90	120	120	60	150
σ_t_ [MPa]	5	5	3	7	6	5	6	3	5	7	5	7	7
Penetration [mm]	3	6	2	6	6	4	4	3	5	3	2	4	5
Blade width [mm]	22	16	16	24	20	20	24	24	18	20	24	18	16

No	14	15	16	17	18	19	20	21	22	23	24	25	
σ_c_ [MPa]	180	180	150	90	180	120	60	150	120	120	90	90	
σ_t_ [MPa]	7	4	4	4	3	6	4	6	4	3	3	6	
Penetration [mm]	2	3	6	2	5	5	5	2	4	6	4	3	
Blade width [mm]	22	18	22	20	20	22	24	18	16	18	22	16

Figures 28 and 29 confirm that the positive force on the cutter ring is proportional to the rolling force, which is consistent with the CSM theory. The intermediate stable part of the data is intercepted and collected, and the average calculated resultant force is taken as the simulation result. The calculation results for CSM can be obtained by substituting the rock and cutter ring design parameters into (4) in Section Cutter ring rock breaking mechanism. Since the cutter spacing parameter S does not exist in the single cutter test, it is assumed that the adjacent cutter has no influence on the research object; hence, S = 200 mm is set. Figure 32 compares the two results.

#### Three-cutter simulation

**Figure 30:**
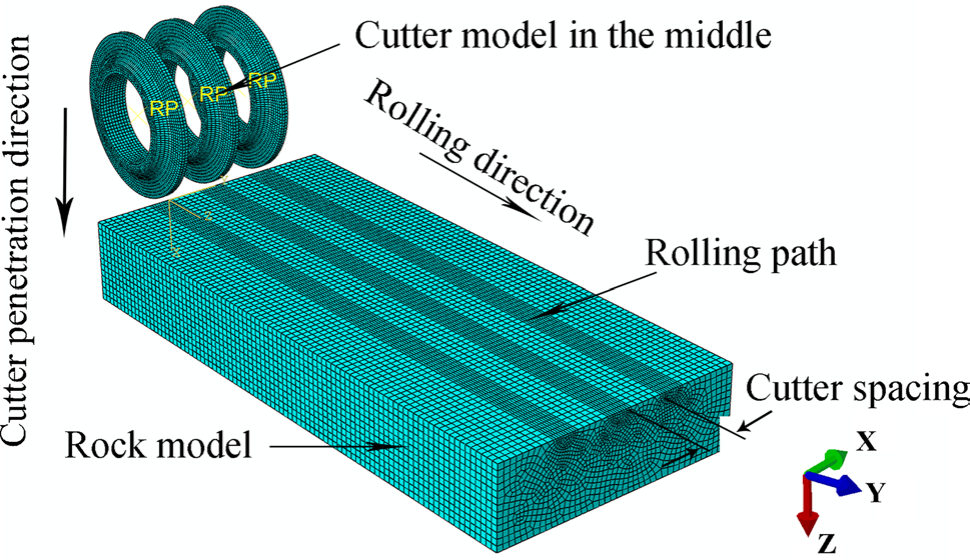
Three-cutters rolling simulation diagram.

The motion model was established for the three-cutter wheel case, as shown in Fig. 30, similar to the model establishment method in Section Penetration simulation; aside from setting three grid subdivisions on the rock-like model surface, the centerline spacing was 80 mm. Three cutter rings were assembled, ensuring that the cutter ring model axes on both sides coincided with the center ring, that the symmetry plane coincided with the grid subdivision area centerline on both sides, and that the middle cutter ring was 1800 mm from the rock in the negative Y direction. The time for the second analysis step = 80 s.

**Figure 31:**
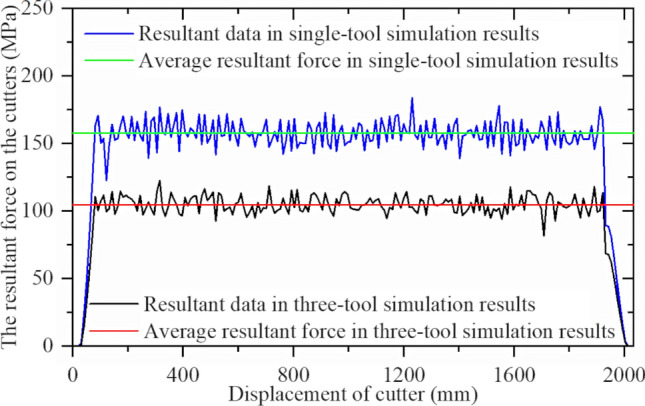
Comparison of the resultant force results of three-cutter simulation and single-cutter simulation.

**Figure 32:**
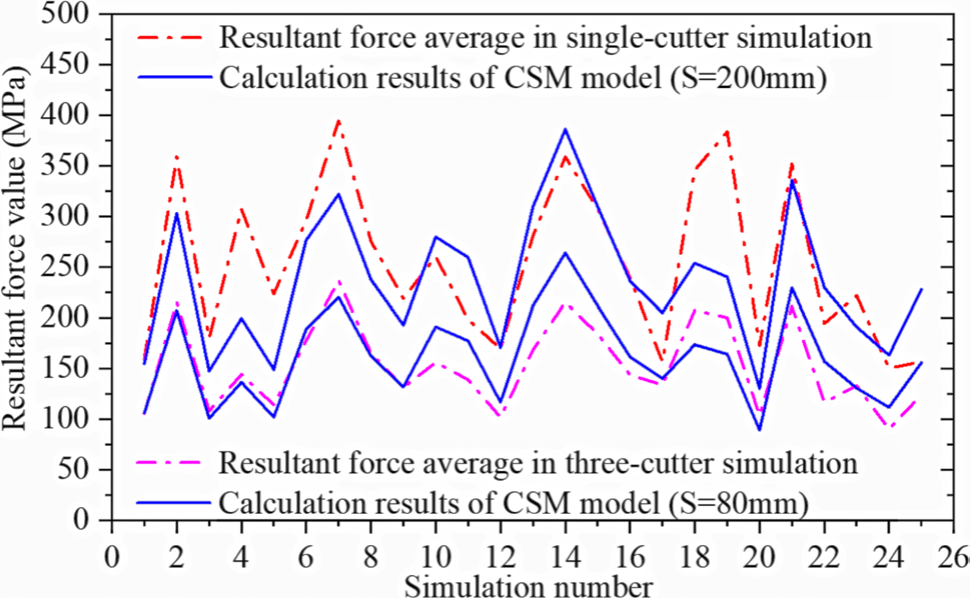
Real and simulated resultant force for CSM models.

A simulation was performed with the middle cutter ring model as the research object, and the resultant force was compared with the single-cutter simulation results. The first group of tests is shown in Fig. 31. The method used to obtain simulation results and CSM calculation results is the same as that used for the single-cutter test above, and Fig. 32 compares the outcomes.

The simulation results are similar to the theoretical calculations, and a reasonable cutter spacing can reduce the force on the cutter ring, hence reducing the energy consumed by the cutter ring. This finding is similar to that of previous studies^2,38,39^, which verifies the validity of the simulation model.

## Specific energy in the simulation results

### Specific energy of the cutter

The macroscopic specific energy is a key parameter for measuring the TBM efficiency and is defined as the ratio of the energy consumed by the TBM to the amount of digging:

22 $${\text{SE}} = \frac{{\text{Q}}}{{\text{A}}},$$

where Q [kWh] is the electrical energy consumed and A [m^3^] is the volume of broken rock.

The specific energy in this study is part of the macroscopic specific energy, which is reflected in the ratio of the kinetic energy consumed by a single disc cutter to the volume of destroyed rock,

23 $${\text{SE}}^{\prime } = \frac{{{\text{Q}}^{\prime } }}{{\text{A}}},$$

where Q' [J] is the kinetic energy consumed by the cutter,

24 $${\text{Q}}^{\prime } = {\text{FL}},$$

where F [kN] is the force on the cutter ring, which can be directly extracted in the simulation, and L [m] is the rolling distance, which can be set in the modeling process. A can also be extracted directly from the simulation result; hence, F, L and A are all known quantities in the simulation result, and ES' can be calculated.

A comparison of the simulation outcomes of CCSs and sinusoidal VCSs for the same rock-like material will clarify which cutter ring has better rock breaking performance. Therefore, the CCS cutter ring was replaced with a sinusoidal VCS cutter ring with different design parameters based on the simulation models above, and 25 sets of single-cutter simulation tests and 25 sets of three-cutter tests were conducted.

### Single-cutter simulation comparison

The two key design parameters for the sinusoidal VCS cutter ring (N and A) were set to 5 values and combined with Table 3 to obtain 25 groups of values. The sinusoidal VCS model was established as described in Section Design of the sinusoidal VCS cutter ring, and the results are shown in Table 4. The CCS cutter ring was successively replaced with the corresponding sinusoidal VCS cutter ring for simulation tests in the single-cutter test scheme in Section Penetration simulation.

**Table 4:** Orthogonal test results for sinusoidal VCS cutter simulations.

No	1	2	3	4	5	6	7	8	9	10	11	12	13
σ_c_ [MPa]	60	180	60	90	60	150	180	150	90	120	120	60	150
σ_t_ [MPa]	5	5	3	7	6	5	6	3	5	7	5	7	7
Penetration [mm]	3	6	2	6	6	4	4	3	5	3	2	4	5
Blade width [mm]	22	16	16	24	20	20	24	24	18	20	24	18	16
N	4	6	2	4	5	3	2	6	2	2	5	6	5
A [mm]	1.5	2.5	1	1	3	1	1.5	3	3	2.5	2	2	1.5

No	14	15	16	17	18	19	20	21	22	23	24	25	
σ_c_ [MPa]	180	180	150	90	180	120	60	150	120	120	90	90	
σ_t_ [MPa]	7	4	4	4	3	6	4	6	4	3	3	6	
Penetration [mm]	2	3	6	2	5	5	5	2	4	6	4	3	
Blade width [mm]	22	18	22	20	20	22	24	18	16	18	22	16	
N	3	5	2	6	4	6	3	4	4	3	5	3	
A [mm]	3	1	2	1.5	2	1	2.5	2.5	3	1.5	2.5	2	

**Figure 33:**
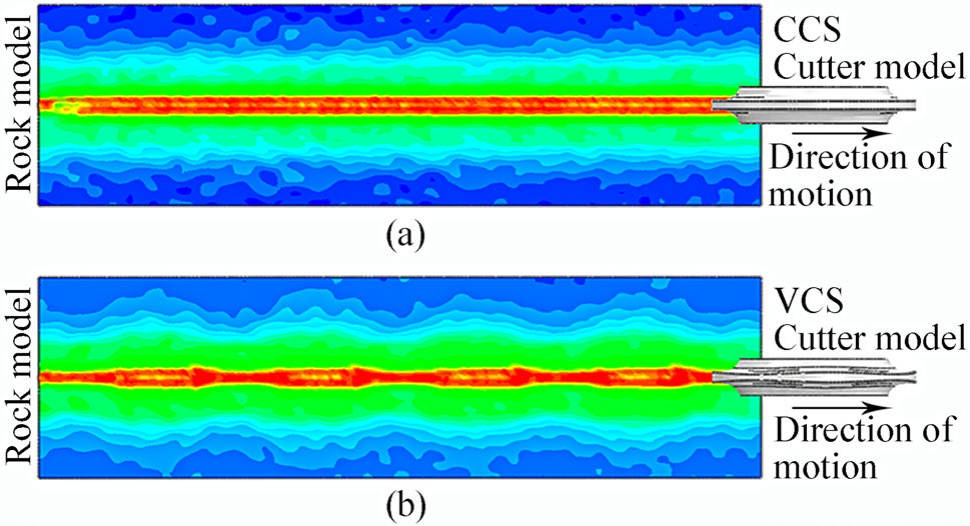
Strain cloud maps for rock-like material elements generated by two cutter rings in single-cutter simulation: (**a**) CCS; (**b**) Sinusoidal VCS.

Figure 33 shows the displacement trend cloud map for the rock-like model element generated by the two cutter ring shapes under the same working environment and basic cutter parameters.

The sinusoidal VCS cutter ring has a fluctuating effect on the rock-like model, and the design objectives described in article 2.2 are realized from this phenomenon.

**Figure 34:**
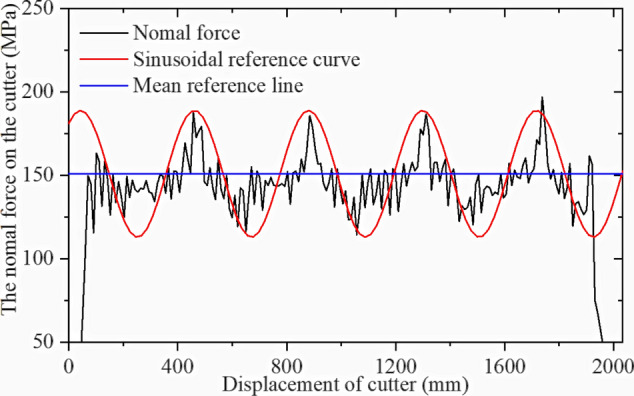
Simulation result for sinusoidal VCS single-cutter rolling, normal force‒displacement curve.

**Figure 35:**
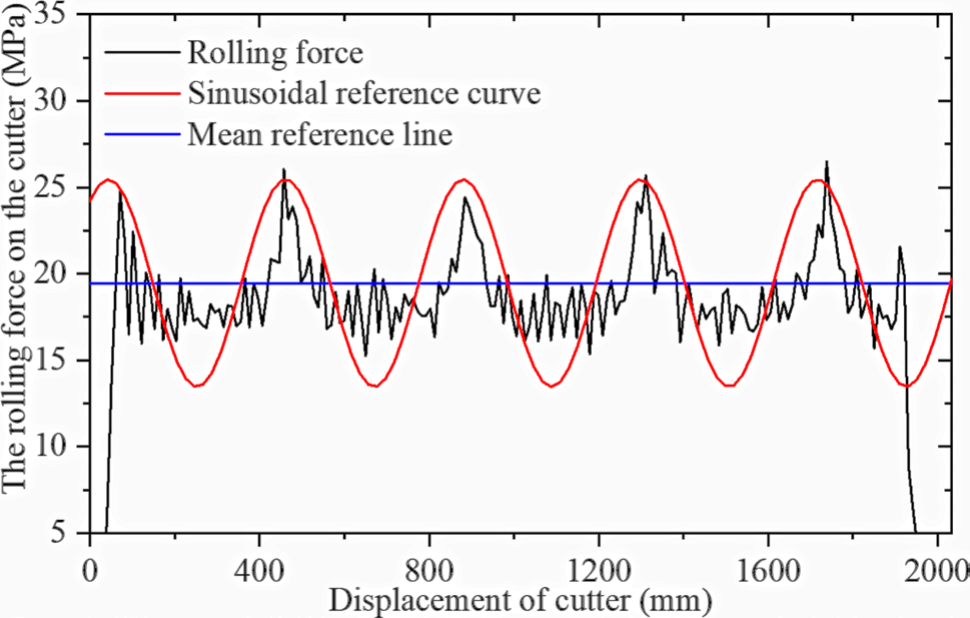
Simulation result for sinusoidal VCS single-cutter rolling, rolling force‒displacement curve.

Figures 34 and 35 show the VCS cuter ring stress results for the first set of results extracted from the simulation. The sinusoidal VCS cutter ring is subjected to fluctuating force, and the fluctuation characteristics correspond to the cutter ring design characteristics, which further verifies that the sinusoidal VCS can achieve the design goal at the principal level.

**Figure 36:**
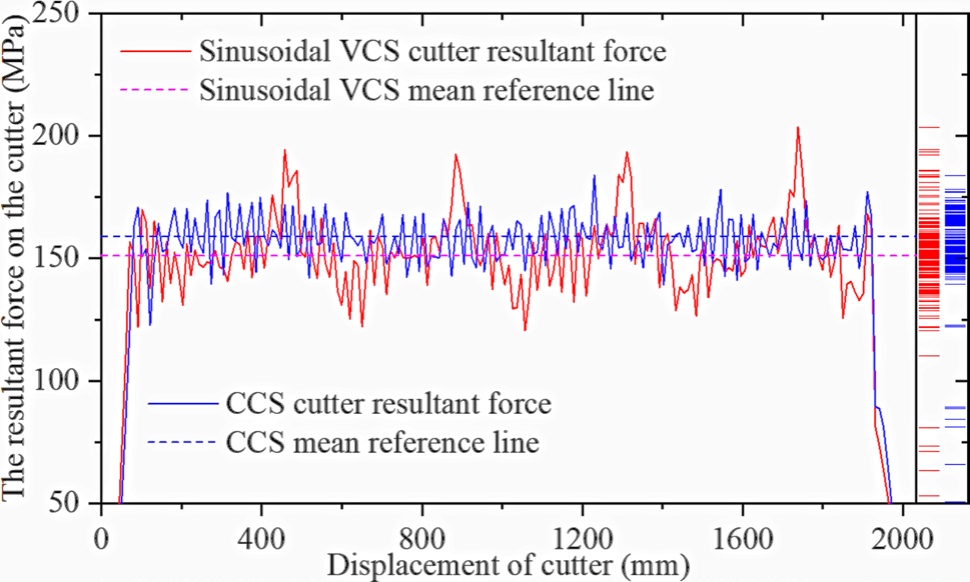
Sinusoidal VCS cutter ring and CCS cutter ring forces in single-cutter simulation.

**Figure 37:**
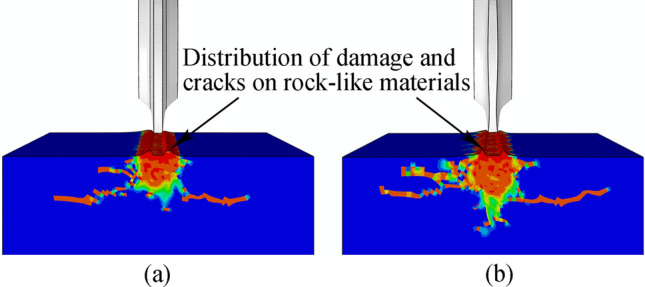
Crack comparison between cutter ring types on rock-like materials: (**a**) CCS cutter; (**b**) sinusoidal VCS cutter.

Figure 36 shows the force and displacement curves for the sinusoidal VCS cutter and CCS cutter rings. For the same displacement, the force on the sinusoidal VCS cutter ring is less than that on the CCS cutter ring. Thus, the VCS cutter ring consumes less energy in the process of rolling rocks, possibly because the fluctuating tensile stress results in more rock damage. To help verify this outcome. Figure 37 compares the rock-like material damage conditions for the simulation and mechanical tests. A sinusoidal VCS cutter ring causes more transverse cracks to form in the rock, promoting crack development and reducing the energy consumed by rolling.

**Figure 38:**
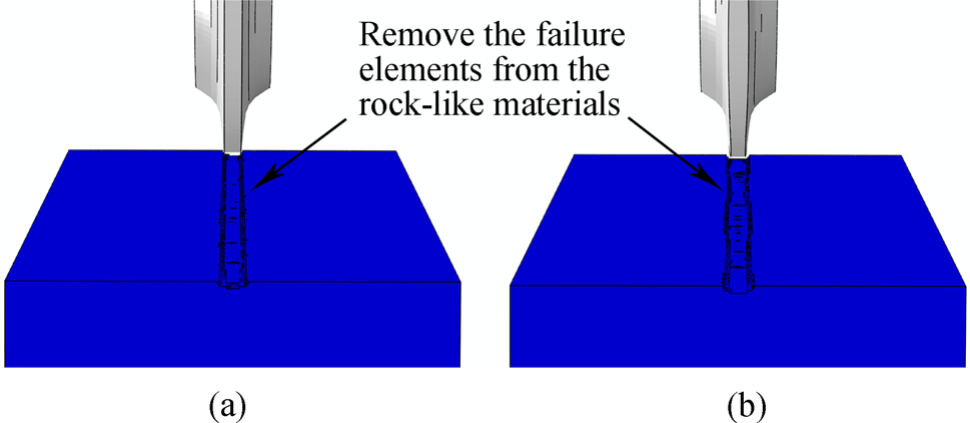
Comparison of Crushing area compared between two cutter ring types on rock-like materials: (**a**) CCS cutter; (**b**) sinusoidal VCS cutter.

**Figure 39:**
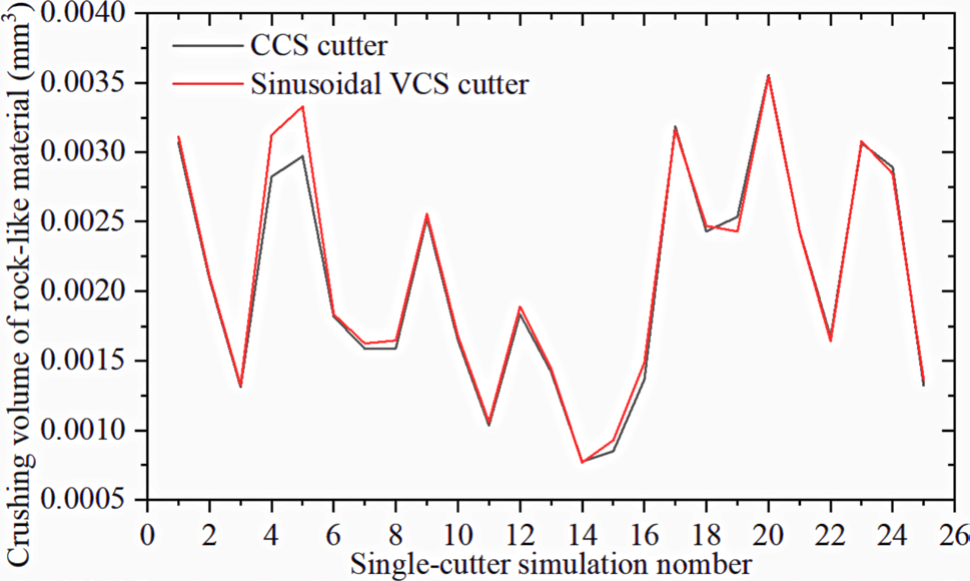
Rock crushing volume caused by CCS and sinusoidal VCS cutters over 25 single-cutter simulations.

Figure 38 compares the crushing effects of two cutter rings on rock-like materials. Sinusoidal VCS blade ring crushing removes more rocks, which is confirmed by the volume reduction data shown in Fig. 39.

**Figure 40:**
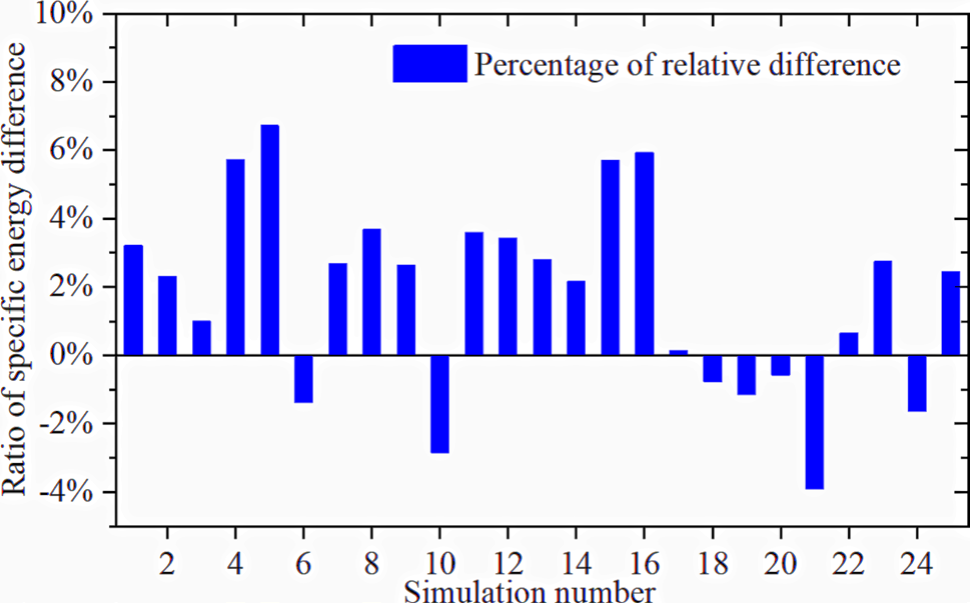
Specific energy differences between sinusoidal VCS and CCS cutter rings in single-cutter simulation test for crushing rock-like materials.

The specific energy for the cutter ring in the 25 groups of single-cutter simulations can be obtained by calculation, as shown in Fig. 40. A reduced specific energy implies improved performance, which can be expressed as

25 $${\text{J}} = \frac{{{\text{SE}}{\prime}_{{{\text{CCS}}}} - {\text{SE}}{\prime}_{{{\text{VCS}}}} }}{{{\text{SE}}{\prime}_{{{\text{CCS}}}} }}{*}100{\text{\% }}{.}$$

Considering Table 4 and Fig. 40, the sinusoidal VCS cutter ring increases performance by approximately 2% compared with the performance of the CCS cutter ring for single-cutter rock rolling simulations. Hence, a 20-inch (508 mm) cutter ring with a 20 mm blade width was used for the rolling simulation experiment with 6 mm penetration. The sinusoidal VCS cutter ring (N = 5, A = 3) achieved a maximum performance improvement of ≈ 7%.

### Three-cutter simulation

The three-cutter simulation comparison is similar to the single-cutter comparison. According to the parameters in Table 4, the CCS was replaced with the corresponding sinusoidal VCS cutter ring in the three-cutter simulation model derived in Section Single-cutter simulation.

**Figure 41:**
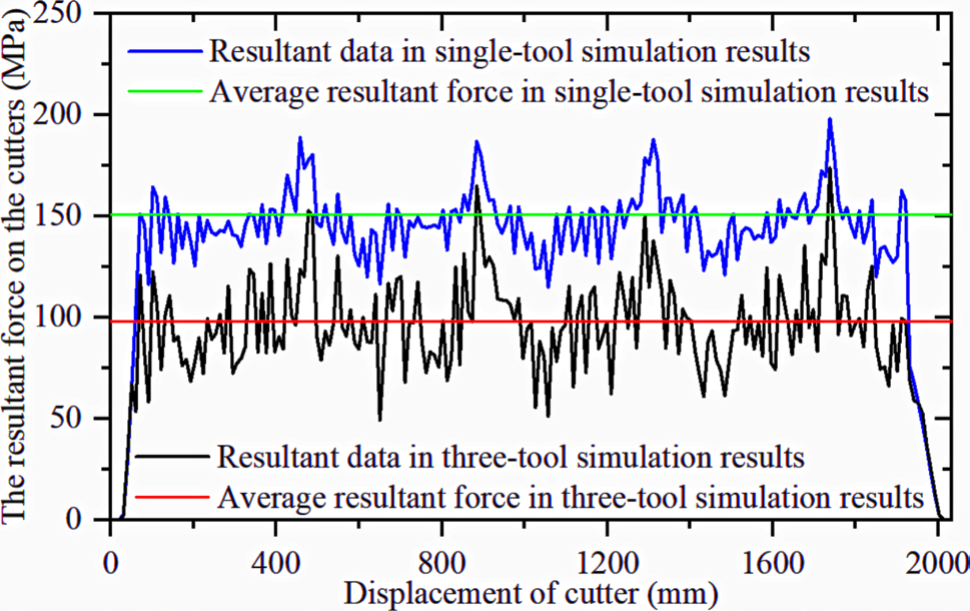
Sinusoidal VCS resultant force for three-cutter and single-cutter simulations.

Figure 41 compares the force‒displacement results for the sinusoidal VCS cutter ring in the single-cutter simulation and three-cutter simulations. The force for the middle cutter decreases when the sinusoidal VCS cutter ring is used, although the fluctuation amplitude increases due to unstable crack development.

**Figure 42:**
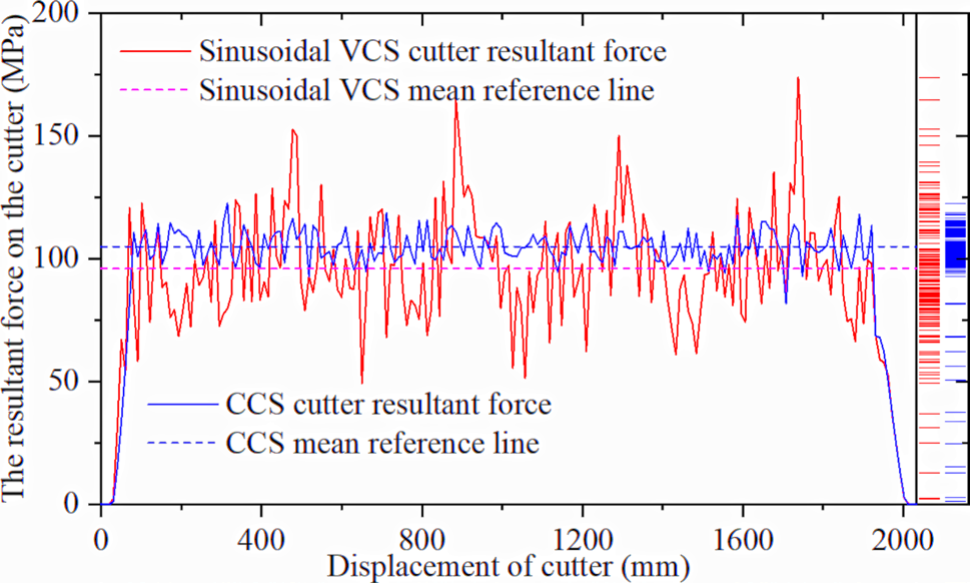
Sinusoidal VCS and CCS cutter rings for the three-cutter simulation.

Figure 42 compares the two cutter ring types in the three-cutter simulation. The sinusoidal VCS cutter experiences less force for the same displacement, further supporting that some sinusoidal VCSs consume less energy to break rock.

**Figure 43:**
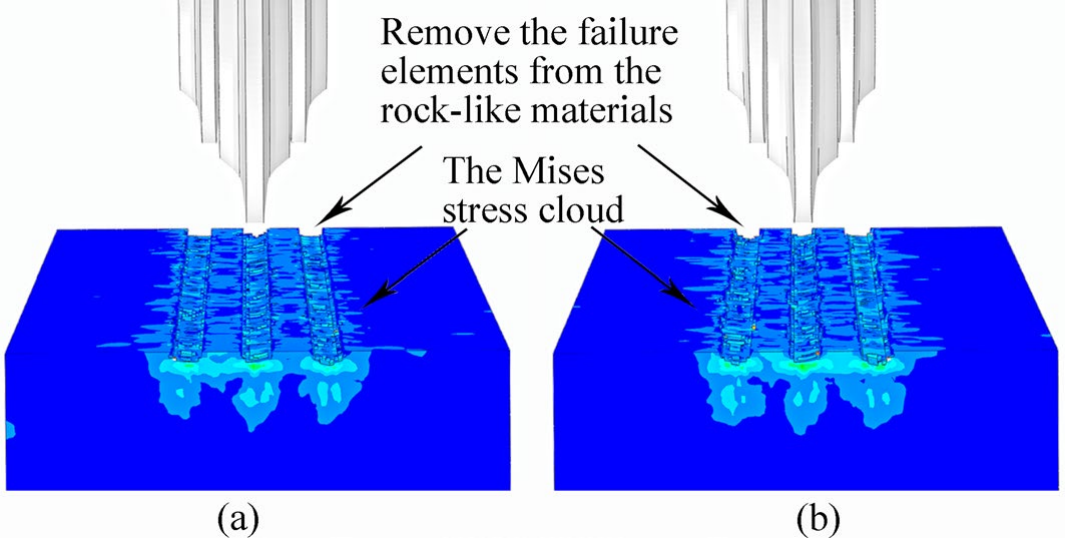
Crushing area and stress nephogram for rock-like material due to (**a**) CCS and (**b**) sinusoidal VCS cutter rings in three-cutter simulation.

Figure 43 compares the damage volume and residual stress cloud map for the two cutter types on the rock-like model. The rock damage volume from the sinusoidal VCS cutter ring is significantly greater than that from the CCS cutter ring, and the stress influence range is significantly greater.

**Figure 44:**
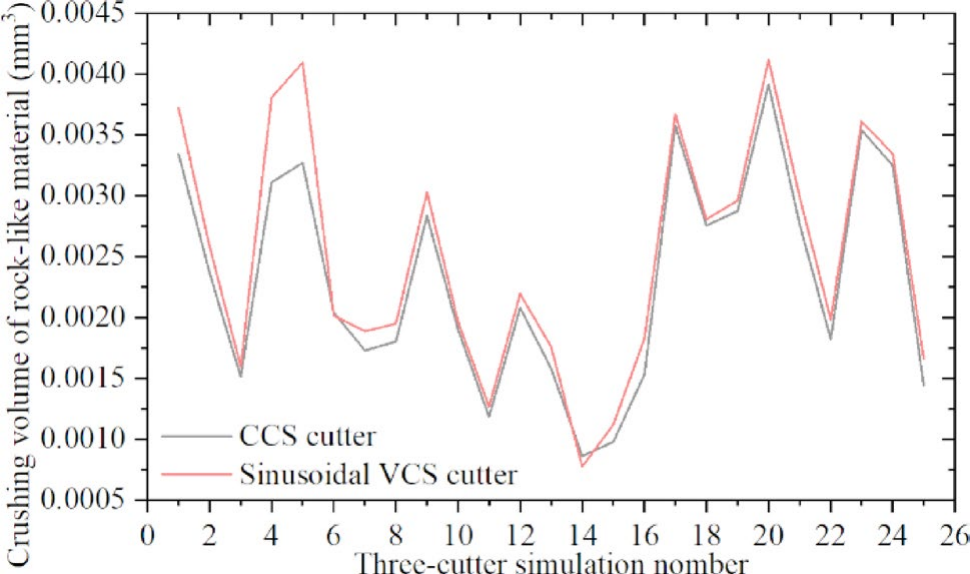
Rock crushing volume due to sinusoidal VCS and CCS cutter rings in 25 sets of three-cutter simulations.

Figure 44 compares the rock breakage volumes for the two cutter types. Most sinusoidal VCS cutters in the figure destroy larger rock volumes than does the corresponding CCS cutter ring.

**Figure 45:**
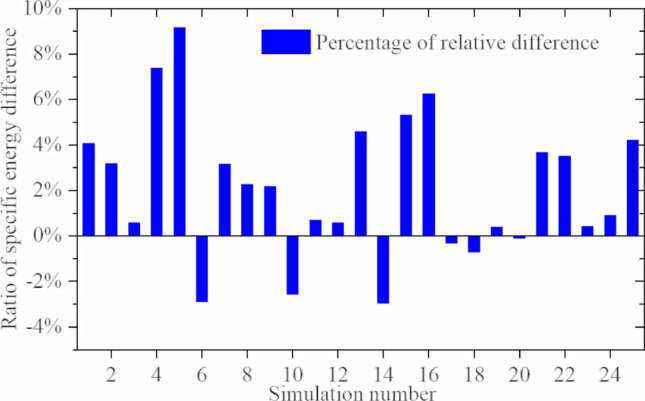
Specific energy differences between sinusoidal VCS and CCS cutter rings in three-cutter simulation for crushing rock-like materials.

Figure 45 shows the sinusoidal VCS cutter ring performance improvement calculated from (25) for the three-cutter simulation. Considering Table 4 and Fig. 45, the sinusoidal VCS cutter ring performance increases by approximately 3% compared with that of the CCS cutter ring for the three-cutter rock rolling simulation. For rock with compressive strength = 60 MPa and tensile strength = 6 MPa, using a 20-inch (508 mm) cutter ring blade width = 20 mm in the rolling simulation with penetration = 6 mm, the sinusoidal VCS cutter ring (N = 5, A = 3) achieved a maximum performance improvement ≈ 9%.

## Conclusion

This study proposed a sinusoidal VCS cutter ring based on CCS cutter ring optimization for known rock mechanical characteristics and the cutter ring rock breaking principle. Two calculation methods were proposed for the key design parameters, and parametric rendering was introduced for 3D models.

A rock-like material design method was established based on the CSM damage constitutive model, and UCS and BTS simulation mechanical tests were conducted. A comparison of the model and real measurements reveals that the strength deviation for the rock-like materials was less than 1%, which confirms the validity of the rock-like material model.

A linear cutting machine simulation model was established, providing simulation results close to the calculated results from the theoretical model, verifying the effectiveness of the LCM simulation model.

Single-cutter and three-cutter orthogonal simulations and tests for CCS and sinusoidal VCS cutter rings were designed based on the rock-like material and LCM simulation models. A comparison of the simulation results across a range of realistic parameters showed that some sinusoidal VCS cutter rings consume less energy when breaking rock, with larger broken rock volumes and hence lower specific energy requirements.

Thus, sinusoidal VCS cutter rings with different design parameters have better lithology breaking performance for some specific working environments. Further research and application of this technology will lead to the identification of optimal candidates for improving the working efficiency of TBMs.

## Data Availability

The datasets used and/or analyzed during the current study are available from the corresponding author upon reasonable request.
